# Drivers of Thermal Habitat Use in Turtles Studied Under Semi‐Natural Conditions

**DOI:** 10.1002/ece3.73325

**Published:** 2026-04-07

**Authors:** Emma White, Jana Stupavsky, Brandon T. Hastings, Austin Ray, Miguel A. Carretero, Pierre Moisson, Julien Claude, Scott Glaberman, Ylenia Chiari

**Affiliations:** ^1^ Department of Biological Sciences Virginia Tech Blacksburg Virginia USA; ^2^ South Alabama Center for Business Analytics, Real Estate, and Economic Development University of South Alabama Mobile Alabama USA; ^3^ School of Biosciences University of Nottingham Nottingham UK; ^4^ Department of Biology University of South Alabama Mobile Alabama USA; ^5^ CIBIO, Research Centre in Biodiversity and Genetic Resources, InBIO Universidade do Porto Vairão Portugal; ^6^ BIOPOLIS Program in Genomics Biodiversity and Land Planning, CIBIO Vairão Portugal; ^7^ Departamento de Biologia, Faculdade de Ciências Universidade do Porto Porto Portugal; ^8^ A Cupulatta Turtle Center Ucciani Corse France; ^9^ Institut des Sciences de L'evolution de Montpellier Université de Montpellier, CNRS, IRD Montpellier France; ^10^ Department of Biology, Faculty of Science Chulalongkorn University Bangkok Thailand; ^11^ Centre for Environmental Research and Justice, School of Biosciences University of Birmingham Birmingham UK; ^12^ School of Life Sciences University of Nottingham Nottingham UK

**Keywords:** comparative ecology, ectotherms, ex situ conservation, microclimate modeling, Testudines, thermal behavior, thermal ecology, thermoregulation

## Abstract

Understanding which factors predict species sensitivity to climate change requires comparative studies conducted under standardized conditions. Reptiles are particularly vulnerable to climate shifts due to their reliance on external temperatures to regulate body temperature. As such, available environmental temperatures may influence their behavior as they seek more optimal conditions. In this study, we measured thermal habitat use in 73 individuals across nine turtle species housed under semi‐natural conditions at a single location. Ambient temperatures within each enclosure were also recorded every 20 min for 3 months to determine the range of thermal options available, yielding over 650,000 data points. We then compared experienced habitat temperatures to environmental conditions across the native range of each species. Experienced habitat temperatures generally aligned with native conditions. However, several species—including *Terrapene carolina* , *Chelonoidis denticulata* , and *Indotestudo elongata*—experienced habitat temperatures near the lower limit of what was available in enclosures and showed little individual variation, suggesting limited capacity for behavioral adjustment under future warming. Experienced habitat temperatures differed among species and were influenced by body mass, but not sex. By providing the first large‐scale, cross‐species dataset on experienced habitat temperatures in turtles under standardized conditions, this study offers a framework for assessing thermal vulnerability and adaptive capacity in response to climate change. These findings also inform conservation efforts, including the design of captive environments that reflect species‐specific thermal needs.

## Graphical abstract

We quantify thermal habitat use in nine turtle species by recording environmental temperatures experienced by individuals in semi‐natural settings. Thermal habitat use differed among species and higher temperatures were generally used by larger animals, pointing to behavioral mechanisms that may influence species responses to environmental change. This work underscores the importance of habitat use data for understanding ectotherm ecology under changing climates.

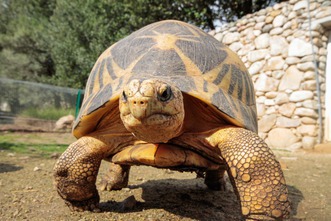

## 1 Introduction

Anthropogenic climate change is driving increases in global temperature and extreme weather conditions (Pachauri and Meyer 2014; Seneviratne et al. 2021), accelerating species extinctions and causing shifts in geographic ranges, phenology, and physiology (Parmesan 2006; Bellard et al. 2012). Ectotherms, such as reptiles, are particularly vulnerable because their internal body temperature is closely linked to environmental conditions (Deutsch et al. 2008). Environmental temperatures influence key biological processes in these organisms, including growth, sex determination, reproduction, digestion, and locomotion (Huey and Stevenson 1979; Huey 1982; Sarre et al. 2004). Consequently, to maintain physiological homeostasis and maximize performance, ectotherms must keep their body temperature within a preferred range (Huey and Stevenson 1979).

Many species of reptiles use microhabitat selection—for example, moving between shade and sun—to maintain body temperatures within the preferred functional range (Bels and Russell 2019). Among reptiles, turtles (Testudines) occupy a broad range of habitats across the aquatic‐terrestrial spectrum (Rhodin et al. 2018; Stanford et al. 2020). They are also among the most threatened vertebrate groups (Rhodin et al. 2018; Stanford et al. 2020), largely due to habitat modification, illegal trade, and climate change (Gong et al. 2009; Stanford et al. 2020). Many turtle species are long‐lived (Mayne et al. 2019; Quesada et al. 2019; Glaberman et al. 2021; Reinke et al. 2022) and have extended generation times (Marsack and Swanson 2009; Scott et al. 2012; Gibbons 1987), which may limit their evolutionary responses to rapid environmental change. Consequently, they may rely heavily on phenotypic plasticity (Curtin 1998; O’Steen 1998; Tamplin and Cyr 2011; Noble et al. 2018; Refsnider et al. 2019) and behavioral thermoregulation (Stillman 2019; Kearney et al. 2009) as more immediate strategies to cope with rising temperatures.

Behavioral thermoregulation in turtles depends on available microhabitat variation (i.e., sun or shade), their familiarity with such variation (Chelazzi and Calzolai 1986), and species‐specific characteristics, including body size, morphology, activity time, sexual dimorphism, and shell thickness (McMaster and Downs 2006; Kearney et al. 2009). Larger‐bodied individuals or species have been shown to retain heat for extended periods and maintain more stable internal body temperatures due to greater thermal inertia (Seebacher and Shine 2004; Polo‐Cavia et al. 2009; Sato 2014; Bulté and Blouin‐Demers 2010, Stevenson 1985). Although ectothermic, turtles with lower surface area‐to‐volume ratios can reduce the rate of heat loss to the environment even in colder climates (Casey et al. 2014; Sato 2014). In contrast, smaller individuals or species gain and lose heat more rapidly and may require more frequent behavioral adjustments, necessitating greater microhabitat thermal variation to maintain their preferred internal temperature (Bulté and Blouin‐Demers 2010).

Although some studies have examined thermoregulatory behavior in turtles (e.g., Perrin and Campbell 1981; Douglass and Layne 1978; Parlin et al. 2017; Wright et al. 1988; Meek 1984), most focus on a single species or even a single population. While such studies are essential for understanding local thermal ranges, they offer limited insight into broader patterns influencing species sensitivity to climatic changes. Comparative studies across multiple individuals and species under similar environmental conditions provide a robust framework for identifying interspecific patterns and assessing the relative importance of factors influencing thermoregulatory behavior and temperature selection in turtles in general (Garland Jr. and Adolph 1994; Garamszegi and Møller 2010). Understanding which factors make species more or less susceptible to climate change is essential not only for conservation and management, but also for exploring the rules shaping species distributions and evolutionary processes (Moore and Schindler 2022).

In this study, we focus on behavioral thermoregulation inferred from thermal habitat use, rather than direct measurements of internal body temperature or thermal performance. Specifically, we tracked the experienced habitat temperatures of nine turtle species and collected environmental data from their semi‐natural enclosures at the “A Cupulatta” turtle sanctuary in Corsica, France. We then compared the experienced habitat temperatures of each species to native microclimate data from their natural ranges. Our goal was to build a comparative dataset of experienced habitat temperatures to assess tolerance to climate variability and extreme temperatures. By examining traits such as body mass, sex, native habitat temperatures, and experienced habitat temperatures under semi‐captive conditions, we aim to understand how these factors influence thermoregulation and shape the capacity of turtles to cope with climate change.

## 2 Materials and Methods

### 2.1 Ethical Approvals

No specific permissions were required prior to conducting this research. The entire research protocol, including the data collection, has been discussed, developed, approved, and carried out under the supervision of the Head of the A Cupulatta center and its staff.

### 2.2 Species Selection and Captivity Conditions

**FIGURE 1:**
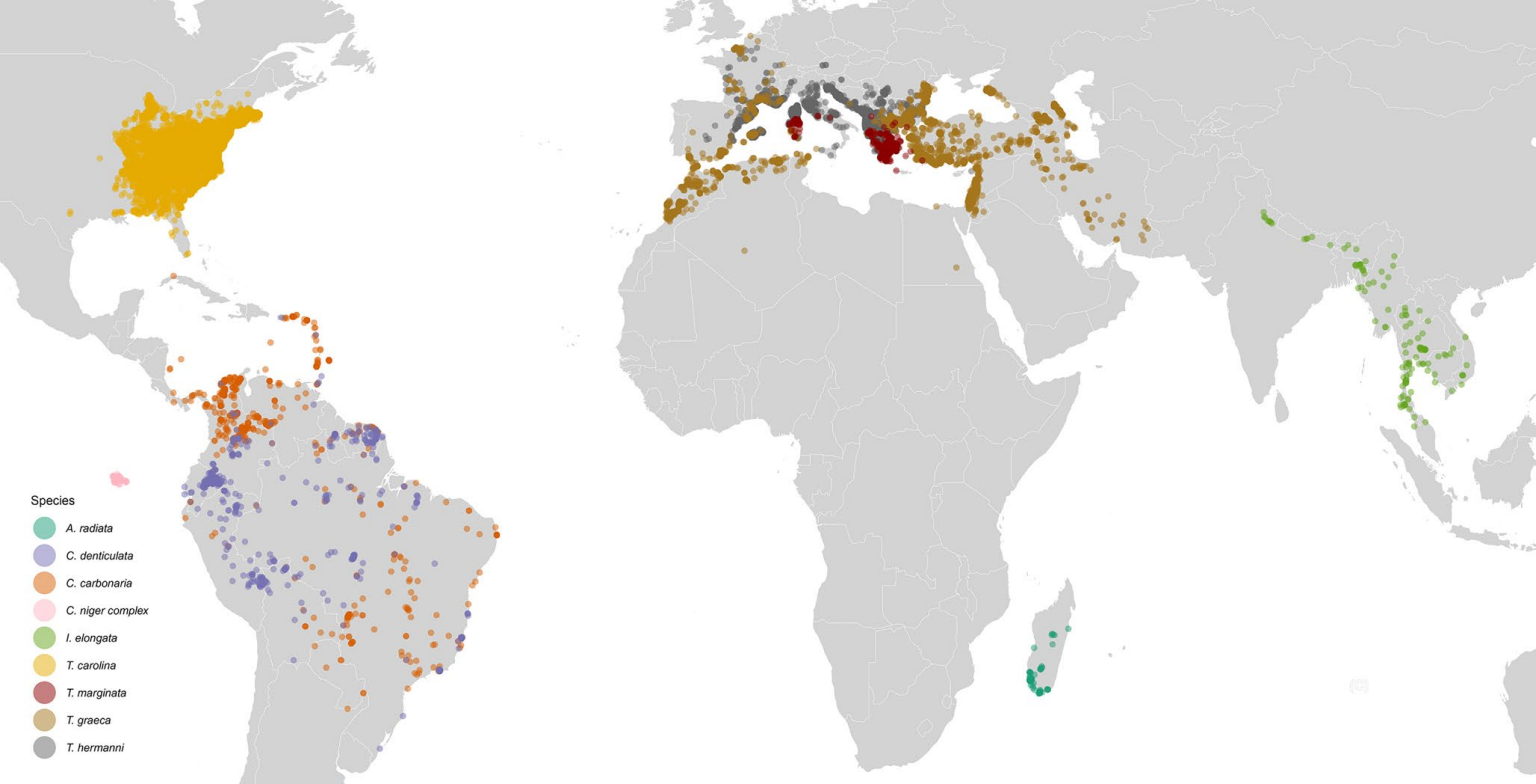
World map of unique Global Biodiversity Information Facility (GBIF) occurrences for each turtle species (GBIF 2022).

We studied nine turtle species at the “A Cupulatta” turtle sanctuary in Corsica, France: *Astrochelys radiata* , *Chelonoidis carbonaria* , *C. denticulata* , *C. niger* species complex, *Indotestudo elongata* , *Testudo graeca* , *T. hermanni* , *T. marginata* (all Testudinidae), and *Terrapene carolina* (Emydidae). Six to ten adult individuals per species were included, except for *C. niger* , for which only two individuals were available (Table 1). Species were selected based on being non‐aquatic (to avoid temperature logger water damage), occurring across diverse climates (Table 1, Figure 1), and having > 5 individuals available. We excluded species showing frequent mating behavior (e.g., *Centrochelys sulcata* ) (Rossi et al. 2023). Only adults were used, as thermoregulatory behavior can vary with age and size (Carretero et al. 1995; Tamplin 2009). When possible, sexes were balanced, and all females were non‐gravid based on breeding history, avoiding potential temperature shifts associated with gravidity (Shine 2012). No mating, nesting, or aggressive behaviors were observed during the study period (July–September). Body mass was measured by A Cupulatta staff, except for *C. niger* , where we used published averages (Chiari 2021) due to their large size (Table 1).

**TABLE 1:** Species and number of individuals per species used in this study (IUCN 2022).

Species	Total number of individuals	Total number of females	Total number of males	Average mass across all individuals (g)	Average shell height (mm)^a^	Climate
*A. radiata*	10	5	5	8350.0	181.0	Tropical
*T. hermanni*	10	5	5	641.3	93.65	Mediterranean
*C. carbonaria*	10	7	3	4846.0	124.5	Tropical
*C. denticulata*	10	7	3	6178.0	149.5	Tropical
*T. marginata*	10	7	3	3196.2	103.6	Mediterranean
*I. elongata*	8	4	4	2254.2	110.0	Tropical
*T. carolina*	7	4	3	477.4	62.3	Temperate
*T. graeca*	6	0	6	672.3	76.86	Mediterranean
*C. niger* species complex	2	0	2	1.42 × 10^5^	592.91	Tropical

*Note:* Species name, total number of individuals per species, number of males and number of females per species, average body mass for each species, average shell height, and native climate category for each species are listed. For *C. niger* , mass was not measured for the two individuals in this study; however, average mass value for this species was obtained as an average across species and sex from published data (Chiari 2021). Native climate category for each species is based on the IUCN Red List for Threatened Species (https://www.iucnredlist.org/). Average shell heights (highest point of the turtle shell) of adult individuals for each species were obtained from the literature.

^a^ If not explicitly specified within each study, average shell height was calculated for each species by averaging measured shell heights across different sexes and populations of adult individuals ( *T. carolina* —Kornilev et al. 2006; *T. graeca* —Tiar‐Saadi et al. 2022; *T. hermanni* —Djordjevic et al. 2011; *C. carbonaria* and *C. denticulata* —Barros et al. 2012; *I. elongata* —Rai 2021; *T. marginata* —Willemsen and Hailey 2003; *C. niger—*Chiari 2021; *A. radiata* —Paquette and Lapointe 2007).

**FIGURE 2:**
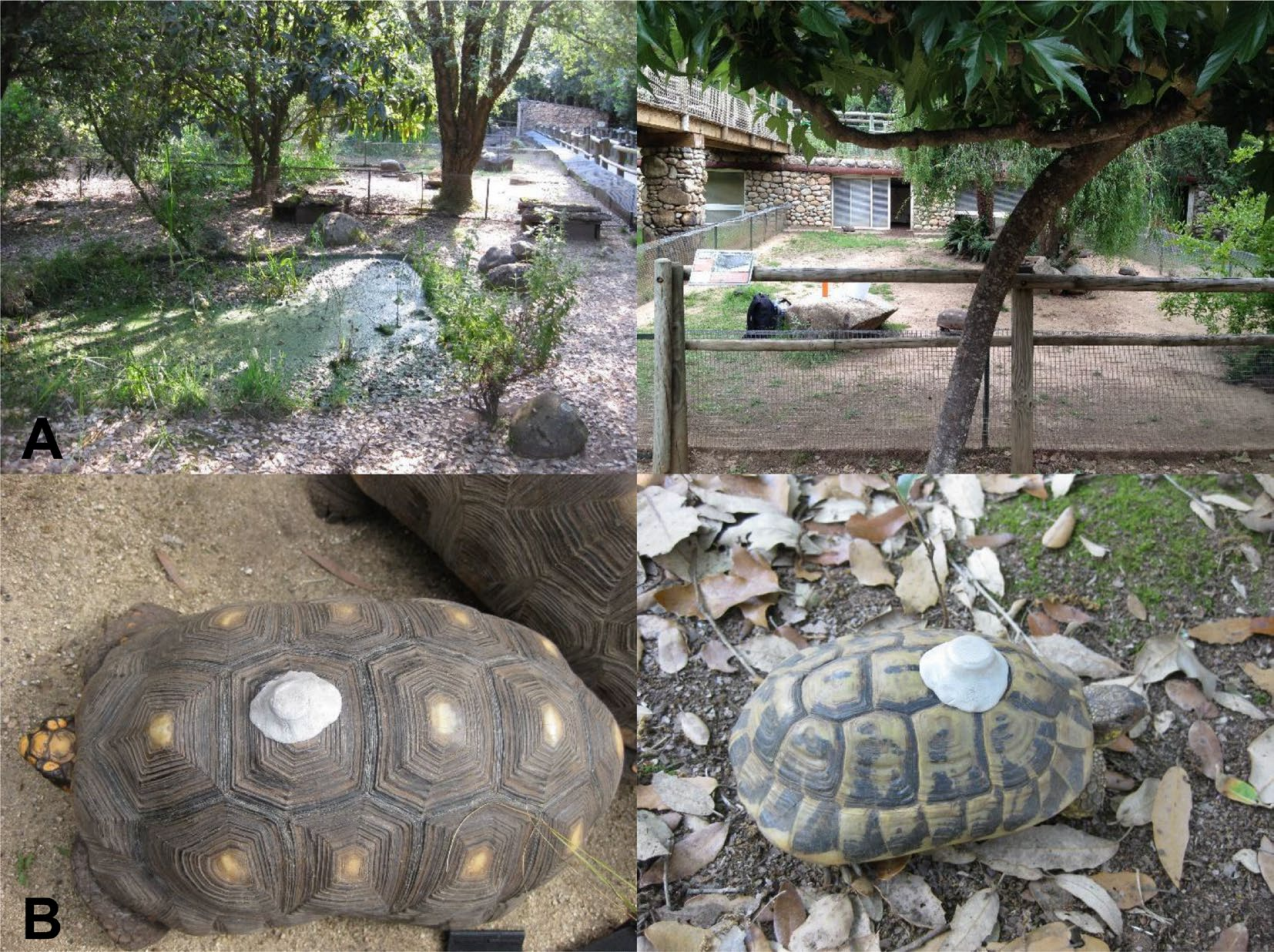
(A) Different turtle enclosures at the A Cupulatta Turtle Sanctuary in Corsica, France. (B) Turtles with i‐buttons secured with epoxy on the carapace of each turtle shell (Left = *C. denticulata* , Right = *T. hermanni* ).

Each species was housed in a separate 165–410 m^2^ enclosure with both sun and shade, natural vegetation, and indoor shelters without climate control (Figure 2). Turtles could freely move between indoor and outdoor spaces. The same food was provided once daily outdoors in fixed locations. Each enclosure also had two water bowls, one indoors and one outdoors, placed in fixed locations.

### 2.3 Data Collection of Experienced Habitat Temperatures for Each Turtle Species

Experienced habitat temperatures only included temperature readings deviating by ±1°C or more from each individual's monthly mean and were defined as the mean of all the temperature readings across the day and season recorded on the carapace of each individual. Experienced habitat temperatures were recorded for each individual by attaching DS1920 iButton data‐loggers (Maxim Integrated, San Jose, CA, USA; ±0.5°C resolution, −55°C to +100°C range) to the second vertebral scute of the carapace using nontoxic epoxy putty, following Bury et al. (2012) (Figure 2). These measurements do not represent internal body temperature, but rather the use of the thermal environments available to the individuals at the study site. To test epoxy effects on temperature readings, we compared loggers with and without epoxy placed side‐by‐side, recording every 30 s over 24 h (*n* = 2821). The epoxy increased recorded temperature by +0.40°C (*t* = 81.29, *p* value < 2.2 × 10^−16^) based on a paired two‐sample *t*‐test, but the values were highly correlated (*r* = 0.996), indicating a consistent difference rather than random variation.

Each logger recorded temperature every 20 min over 90 days (July 2–Sept 30, 2014), starting at 12:01 am. Additional epoxy‐mounted DS1920 iButtons were placed in each enclosure to capture ambient temperatures in sun, shade, and inside the housing structure. One logger per enclosure was placed near the daily food bowl, typically located in full sun. Enclosures for *C. niger* and *T. graeca* had only two environmental loggers (inside and outside) due to low habitat variability, while the other species had three (one inside, two outside) to reflect more variable environments. All loggers were synchronized to record at identical time points across all individuals and enclosures. After the study, devices were removed, cleaned, and data extracted using a DS1402D Blue Dot Receptor (Maxim Integrated).

### 2.4 Data Cleaning

On a few days during the sampling season, A Cupulatta staff confined some species indoors due to low outdoor temperatures. Since movement was restricted and indoor temperatures were relatively uniform, data from these days did not reflect complete thermal ranges (e.g., with no thermal restrictions). Although we did not monitor the exact days in which turtles were confined, we statistically removed such periods by filtering out 24‐h periods where most individuals of a species showed no thermal variation. Specifically, we excluded days where over half the individuals had experienced habitat temperatures within ±1°C of their daily mean, using this conservative threshold to minimize the impact of noise. Filtering was done separately for each species. Only one full day was excluded ( *C. denticulata* on July 9, 2014). Additionally, we removed two outlier days (July 23 and August 6, 2014) for a single *C. denticulata* individual whose recorded temperatures exceeded 50°C—values not supported by enclosure temperature data.

Since behavioral thermoregulation occurs when animals are active and selecting specific temperatures (Huey 1982), we focused our analysis on daylight hours. Nighttime experienced habitat temperatures showed significantly lower variation (mean SD = 1.60) compared to daytime (mean SD = 3.94), confirming inactivity at night (two‐sample *t*‐test, *p* value < 0.05; R Core Team 2021). This aligns with reports that these species are diurnal in the wild (e.g., Meek and Jayes 1982; Fasola et al. 2002; Blake, Parlin, et al. 2021; Blake, Tapia, et al. 2021). We defined activity periods using historical sunrise/sunset data for Tavaco, Corse‐du‐Sud, France (∼1142 m from A Cupulatta): 6:00–21:00 in July, 6:20–20:40 in August, and 7:00–20:00 in September (https://www.timeanddate.com/sun/@2973271?month=7&year=2014).

To isolate data when turtles were actively thermoregulating, we selected temperature readings deviating by ±1°C or more from each individual's monthly mean. These “active” experienced habitat temperatures were used in all subsequent analyses of thermoregulatory behavior.

### 2.5 Data Extraction of Native Microclimate Temperatures for Each Turtle Species

Of the nine turtle species in this study, five are tropical ( *A. radiata* , *C. carbonaria* , *C. denticulata* , *I. elongata* , *C. niger* ), three are Mediterranean ( *T. hermanni* , *T. marginata* , *T. graeca* ), and one is temperate ( *T. carolina* ) (Table 1). To estimate native environmental temperatures, species occurrence data were downloaded from the Global Biodiversity Information Facility (https://www.gbif.org/; March 2023) using the “occ_download_get” function (*rgbif* package; Chamberlain et al. 2014) and cleaned in RStudio (v4.1.2) using “clean_coordinates” (*Coordinate Cleaner*; Zizka et al. 2019). Species occurrences ranged from the year 1800 to 2023 with most coordinates past the year 2000. Records with mismatches, missing values, or invalid coordinates were removed. The few occurrences outside native ranges (based on “countryCode”) were excluded manually. Based on the min and max range of longitudes and latitudes from each cleaned dataset, ERA5 hourly climate data (July–September 2014) were downloaded using the “request_era5” function (*mcera5* package; Klinges et al. 2022) from the Climate Data Store (https://cds.climate.copernicus.eu/). Microclimate temperatures at each coordinate (*n* = 29,709) were estimated hourly with micro_era5 (NicheMapR; Kearney and Porter 2017), using species‐specific midpoint body height (half the shell height) as local model height. Shell height values were obtained from published literature on adult individuals (Table 1). For each coordinate, temperatures were modeled under full sun (0% shade) and full shade (100% shade). In each microclimate model for each species, the parameter “minshade” was set to 0 to estimate temperatures with no vegetation cover for each coordinate (i.e., 0% shade) and the parameter “maxshade” was set to 100 to estimate temperatures with complete vegetation cover for each coordinate (i.e., 100% shade). To align with field sampling, only values from July 2‐Sept 30 2014 during the hours of 6:00 to 21:00 were included in analyses of “active” periods. The full dataset including experienced and enclosure temperatures are available on Dryad. The entire dataset and the R codes used for the analyses can be found on Dryad (https://doi.org/10.5061/dryad.5x69p8dhg). Code for filtering turtle experienced habitat temperature data can be found here: https://github.com/brandon‐hastings/thermal_data_cleaning.

### 2.6 Data Visualization

Dashboards were created using Tableau Desktop 2022.4.4 (https://www.tableau.com/products/desktop) to visualize data on average hourly temperatures for July, August, and September. The data were displayed across five tabs: native environment temperatures, enclosure temperatures, and estimated temperatures for each species and individual. Heatmap charts show the temperature ranges by species or individual, organized by each hour of the day during the sampling period. Users can customize views using a selection tool (if available) and hover over charts for additional details on temperature calculations. The visualizations are available at: https://public.tableau.com/app/profile/jana.stupavsky5766/viz/Turtles_DraftActive_V2/1‐NativeAvgTemps.

### 2.7 Statistical Analyses

Statistical analyses were conducted in RStudio 4.3.3 (R Core Team 2024). Two‐way ANOVA was used to assess the effects of sex and species on log body mass, including their interaction. If significant, a Tukey post hoc test identified pairwise differences in log body mass between species and between males and females within each species. One‐way ANOVA was performed to determine the effects of sex on log body mass separately for species with large mean differences in log body mass between males and females including *T. hermanni* and *T. marginata* . The analyses used the “aov” and “TukeyHSD” functions from the *stats* package (R Core Team 2024).

Native microclimate temperatures extracted from the natural geographic ranges of each species for the period of July to September 2014 were averaged for each individual coordinate during all times of day and during times that turtles within this study were most active (6 am–9 pm; see Section 2.3). Levene's and Shapiro–Wilk tests assessed homoscedasticity and normality of these temperatures across species. Since the data had unequal variances and were not normally distributed, one‐way Welch's ANOVA was used to compare differences in shade temperatures for each species, with a Games‐Howell post hoc test for pairwise comparisons. A Games‐Howell post hoc test was then used to compare significant differences of native microclimate temperatures during “active” time (6 am–9 pm) and across the entire day for each pair of species. These analyses were performed for native sun microclimate data (native temperatures estimated with 0% vegetation cover), native shade microclimate data (native temperatures estimated with 100% vegetation cover) and combined native sun and shade microclimate data. All Welch's ANOVAs were performed using the “oneway.test” function from the *rstatix* package (Kassambara 2023). Games‐Howell post hoc tests were performed using the “games_howell_test” function from the *stats* package. To test if experienced habitat temperatures in each species differed from their native microclimates (sun or shade), two‐sample Wilcoxon rank‐sum tests were performed using the “wilcox.test” function from the *stats* package. For these tests, species experienced habitat temperatures were averaged across all times for all months of the season for each individual. Native microclimate temperatures (sun and shade) from July to September 2014 were averaged for each species at each individual coordinate during all times and “active” time (6 am–9 pm).

To assess differences between experienced “active” temperature and enclosure temperatures (sun or shade) of each species, two‐sample *t*‐tests were manually performed in RStudio separately for each species. Experienced habitat temperatures were averaged across all 20‐min intervals from 6 am to 9 pm, July to September, for all individuals per species. Enclosure temperatures (sun and shade) were averaged the same way. For these tests, we assumed that sun temperatures exceeded experienced values and shade temperatures fell below them. Based on this, differences were calculated accordingly: experienced habitat temperatures minus sun for sun tests, and shade minus experienced for shade tests. *T*‐values were calculated manually using each species' difference, standard deviation of mean experienced habitat temperatures across individuals, and the square root of the sample size for each species, following the formula *sign_difference/(std_dev/sqrt(n))*.

To assess variation in estimated experienced habitat temperatures within and among species, we calculated the average and standard deviation of experienced habitat temperatures recorded every 20 min throughout the season for each individual. Standard deviation was used as a measure of the amount of variation within and across species. One‐way ANOVA was performed to test the effects of species, sex, or log body mass on both average and standard deviation of experienced habitat temperatures across species. For species with 10 individuals ( *C. carbonaria* , *C. denticulata* , *T. hermanni* , *T. marginata* , and *A. radiata* ), a two‐way ANOVA was run separately to test the effects of species and log body mass and species and sex (and the interactions between those factors) on both average and standard deviation of experienced habitat temperatures across all species. Tukey post hoc tests compared pairwise differences across and within species. All analyses were run for both full‐day and “active” time (6 am–9 pm) using the aov and TukeyHSD functions.

## 3 Results

From July 2 to September 30, 2014, we collected 477,432 data points from data loggers secured to 73 individuals across nine turtle species. An additional 180,000 temperature data points were recorded from data loggers placed in the enclosures of each species. In total, 657,432 data points were collected from both turtle and environmental loggers.

### 3.1 Inter‐ and Intra‐Specific Variation in Body Mass

Two‐way ANOVA and Tukey post hoc tests showed significant differences in log body mass among species (*p* value < 0.001), but no statistically clear differences were shown for log body mass between males and females within or among species (*p* values > 0.05) (Table 2, Table S1), with the exception of *T. hermanni* , which had higher body mass in females than males (one‐way ANOVA mean difference = −0.47, df = 1, SS = 0.55, MS = 0.55, *F*‐value = 13.7, *p* value = 0.006).

**TABLE 2:** Influence of species and sex on log body mass as estimated by a two‐way ANOVA.

Variable	df	SS	MS	*F*	*p*
Log body mass					
Species	8	106.96	13.37	130.73	**< 2.0 × 10** ^**−16**^
Sex	1	0.03	0.03	1.58	0.56
Species:Sex	6	0.97	0.16	1.58	0.17

*Note:* *p* values in bold represent statistical significance (< 0.05) (df, degrees of freedom; MS, mean squares; SS, sum of squares). *C. niger* and *T. graeca* were included in the analyses. See also Table S1 for results of Tukey post hoc test.

### 3.2 Interspecific Differences in Native Microclimate Temperatures

**FIGURE 3:**
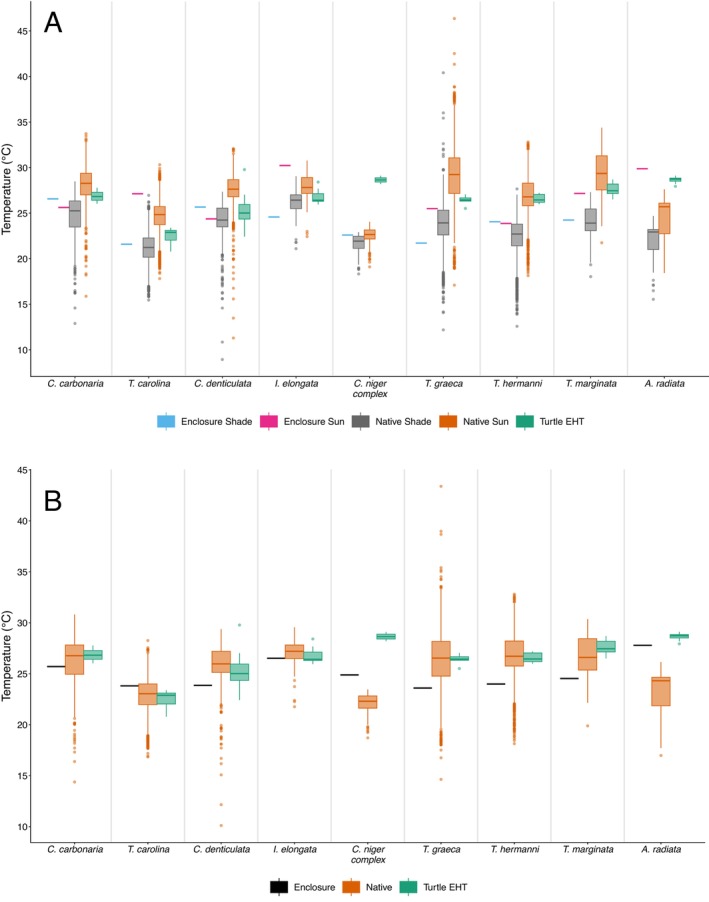
Native microclimate temperatures (averaged across the season for each species' coordinates), averaged enclosure temperatures (across the study season), and experienced habitat temperatures (averaged across the study season for each individual) (in °C) across turtle species during “active” time (from sunrise to sunset) for July–September. Colors correspond to enclosure, native, and turtle as indicated in the figure legend. (A) Shade and sun native and enclosure temperatures are graphed separately. (B) Data on shade and sun combined for the enclosure data and for the native temperatures. For *C. carbonaria* , *C. denticulata* , *A. radiata* , and *C. niger* native temperatures from July–September correspond to the lower temperatures experienced during the year. Turtle EHT = turtle experienced habitat temperature.

During their “active” time (6:00 am to 9:00 pm) from July to September, turtle species differed in native sun, native shade, and combined native sun and shade microclimate temperatures (Welch's ANOVA; *p* values < 0.05 for all tests; Table 3, Figure 3, and data visualization Table 1). Games‐Howell post hoc tests supported significant pairwise differences in native sun, native shade, or combined native sun and shade between most pairs of species (*p* values < 0.05) with a few exceptions (Tables S2–S4). Differences in native microclimate temperatures (sun, shade, or combined) were not statistically clear between *T. graeca* and *T. marginata* (Tables S2–S4). All the results were confirmed when Welch's ANOVA was repeated using the entire day instead of only the “active” time (Tables S5–S8).

**TABLE 3:** Effect of species on native sun, shade, and combined shade and sun microclimate temperatures during “active” time (6 am–9 pm) for months July–September.

Source	dfn	dfd	*F*	*p*
Native sun temp				
Species	8	814.65	2177.5	**< 2.2 × 10** ^**−16**^
Native shade temp				
Species	8	814.67	1302	**< 2.2 × 10** ^**−16**^
Native sun & shade temp				
Species	8	814.44	3633.9	**< 2.2 × 10** ^**−16**^

*Note:* *p* values in bold represent statistical significance (< 0.05).

### 3.3 Comparison of Experienced Habitat Temperatures Versus Native Microclimate Temperatures and Enclosure Temperatures

Wilcoxon rank sum tests indicated no statistically clear differences between combined native sun and shade microclimate temperatures compared to “active” experienced habitat temperatures for each species (*p* values > 0.05), except for *A. radiata* , which experienced habitat temperatures 5.36°C higher than native microclimates (*p* value < 0.001) during “active” time (Table 4, Figure 3, and data visualization Table 2). In contrast, combined native sun and shade microclimate temperatures significantly differed from experienced habitat temperatures across the entire day for all species (*p* values < 0.05) except for *T. graeca* (mean difference = −0.07°C, *p* value = 0.75) (Table S9).

**TABLE 4:** Two‐sample Wilcoxon rank‐sum test comparisons of experienced habitat temperatures versus native microclimate temperatures (sun, shade, and sun and shade combined) during “active” time by species.

Species	Native microclimate	Mean difference (°C)	95% CI (lower, upper)	*W*	*p*
*C. denticulata*	Sun	−2.09	(−3.71, −1.26)	979	**0.001**
	Shade	1.23	(−0.27, 1.95)	2935	0.13
	Both	−0.43	(−2.01, 0.36)	1650	0.12
*A. radiata*	Sun	4.03	(2.66, 5.07)	820	**2.79 × 10** ^**−7**^
	Shade	6.70	(5.57, 7.18)	820	**2.79 × 10** ^**−7**^
	Both	5.36	(4.12, 6.25)	820	**2.79 × 10** ^**−7**^
*C. carbonaria*	Sun	−1.21	(−2.26, −0.49)	1503	**0.005**
	Shade	2.12	(0.75, 2.67)	5088	**4.73 × 10** ^**−4**^
	Both	0.45	(−0.71, 1.09)	3214	0.83
*I. elongata*	Sun	−1.09	(−1.97, −0.36)	230	**0.01**
	Shade	0.62	(−0.39, 1.34)	607	0.31
	Both	−0.23	(−1.13, 0.43)	374	0.23
*T. graeca*	Sun	−2.75	(−4.50, −0.90)	3694	**0.006**
	Shade	2.52	(1.18, 3.74)	18,081	**0.001**
	Both	−0.11	(−1.65, 1.40)	9706	0.84
*T. marginata*	Sun	−1.77	(−3.18, −0.41)	1044	**0.008**
	Shade	3.50	(2.59, 4.33)	4065	**1.08 × 10** ^**−7**^
	Both	0.86	(−0.24, 1.94)	2628	0.12
*T. hermanni*	Sun	−0.35	(−1.13, 0.50)	27,182	0.58
	Shade	3.98	(3.18, 4.68)	60,400	**4.77 × 10** ^**−8**^
	Both	−0.27	(−1.06, 0.58)	28,219	0.71
*T. carolina*	Sun	−2.21	(−3.20, −1.26)	14,339	**3.68 × 10** ^**−4**^
	Shade	1.27	(0.33, 2.29)	99,777	**0.01**
	Both	−0.47	(−1.41, 0.50)	49,837	0.29

*Note:* *p* values in bold represent statistical significance (< 0.05). (Mean difference = experienced avg.—native microclimate temperature avg. (sun, shade or both), CI, confidence interval; W, Wilcoxon rank‐sum value). *Chelonoidis* sp. was not included in this analysis as only two individuals could be studied. Note that significant comparisons were unlikely to be driven by the effect of epoxy, which increased readings by 0.41°C; all significant differences exceeded 0.41°C.

Wilcoxon rank‐sum tests revealed that during “active” time, experienced habitat temperatures tended to be significantly lower than native sun microclimate temperatures (*p* values < 0.05) with the exception of *A. radiata* , which experienced habitat temperatures 4.03°C higher than native sun microclimates (*p* value < 0.001) (Table 4) and *T. hermanni* , which showed no statistically clear difference (mean difference = −0.35°C, *p* value = 0.58) (Table 4). Native shade microclimate temperatures were significantly lower than experienced habitat temperatures for all species with the exceptions of *C. denticulata* (mean difference = 1.23°C, *p* value = 0.13) and *I. elongata* (mean difference = 0.62°C, *p* value = 0.31) (Table 4), which both showed no statistically clear differences. These results were generally confirmed for all times during the day except that *T. marginata* experienced habitat temperatures did not differ from native sun (mean difference = −0.40°C, *p* value = 0.43) and *A. radiata* , *T. graeca* , *T. marginata* , and *T. hermanni* exhibited higher experienced habitat temperatures than native shade microclimates (*p* values < 0.05) (Table S9). Comparisons between experienced habitat temperature, native microclimate temperature, and enclosure temperature means can be viewed under Data Visualization (Section 2.5, data viz. Table 3).

Two‐sample *t*‐tests did not show statistically clear differences between sun enclosure temperatures and species experienced habitat temperatures during “active” time for all species, except for *T. carolina* , *A. radiata* , and *I. elongata* (*p* values < 0.05, Table 5). Sun enclosure temperatures tended to be higher than experienced habitat temperatures for these three species (*p* values < 0.05, Table 5). These results were generally confirmed when using data for all times of day, except for *A. radiata* (*p* value = 1.0, Table S10). For all species except for *C. denticulata* , species experienced habitat temperatures were significantly higher than shade enclosure temperatures during “active” time (*p* values < 0.05, Table 5). When using data for all times of day, experienced habitat temperatures for all species, including *C. denticulata* , were significantly higher than shade enclosure temperatures (*p* values < 0.05, Table S10).

**TABLE 5:** Two‐sample *t*‐test comparisons of experienced habitat temperatures and enclosure temperatures (sun or shade) during “active” time for each species.

Species	Enclosure microclimate	Mean difference (^o^C)	95% CI (Lower, Upper)	SE (^o^C)	df	*t*	*p*
*T. carolina*	Sun	4.13	(3.25, 5.02)	0.36	6	11.42	**1.34 × 10** ^**−5**^
	Shade	1.16	(0.28, 2.05)	0.36	6	3.22	**0.008**
*C. denticulata*	Sun	−1.50	(−2.29, −0.007)	0.66	9	−2.27	0.97
	Shade	0.14	(−1.34, 1.64)	0.66	9	0.22	0.41
*A. radiata*	Sun	1.03	(0.77, 1.28)	0.11	9	9.10	**3.87 × 10** ^**−6**^
*C. carbonaria*	Sun	−1.48	(−1.92, −1.05)	0.19	9	−7.71	0.99
	Shade	0.70	(0.26, 1.14)	0.19	9	3.65	**0.002**
*I. elongata*	Sun	2.98	(2.27, 3.68)	0.29	7	10.00	**1.06 × 10** ^**−5**^
	Shade	2.60	(1.90, 3.13)	0.29	7	8.75	**2.54 × 10** ^**−5**^
*T. graeca*	Sun	−0.85	(−1.40, −0.31)	0.21	5	−4.06	0.99
	Shade	4.76	(4.22, 5.31)	0.21	5	22.59	**1.57 × 10** ^**−6**^
*T. hermanni*	Sun	−2.76	(−3.10, −2.42)	0.15	9	−18.28	1.0
	Shade	2.57	(2.03, 3.12)	0.15	9	17.04	**1.85 × 10** ^**−8**^
*T. marginata*	Sun	−0.80	(−1.32, −0.29)	0.22	9	−3.56	0.99
	Shade	3.65	(3.14, 4.16)	0.22	9	16.08	**3.06 × 10** ^**−8**^
*C. niger* complex	Shade	6.82	(0.99, 12.65)	0.45	1	14.88	**0.02**

*Note:* *p* values in bold represent statistical significance (< 0.05).

Abbreviations: CI, confidence interval; df, degrees of freedom; Mean difference (shade), experienced habitat temperature avg.—shade enclosure temperature avg.; Mean difference (sun), sun enclosure temperature avg.—experienced habitat temperature avg.; SE, standard error.

### 3.4 Inter‐ and Intra‐Specific Differences in Average Experienced Habitat Temperatures

**FIGURE 4:**
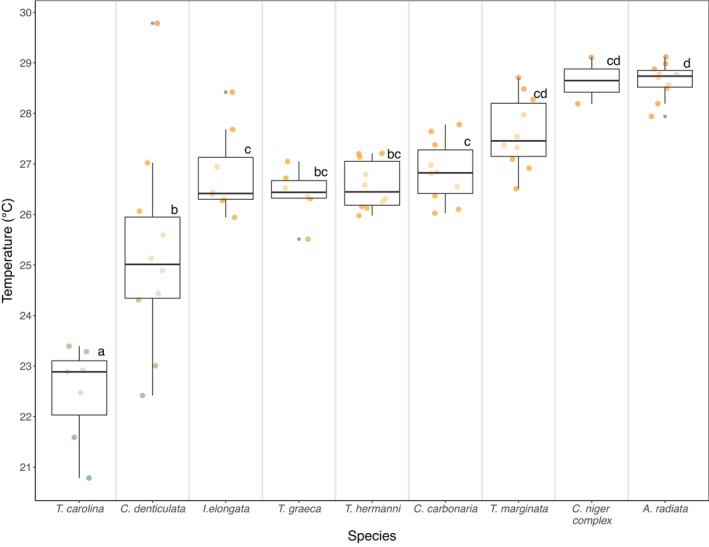
Average experienced habitat temperatures across nine turtle species for each individual during the “active” time across the entire season. Letters shared above boxplots for each species denote no statistically clear differences in “active” average experienced habitat temperature. Letters not shared between boxplots for each species denote significant differences in experienced “active” average temperature based on the Tukey post hoc test (Section 3.4; Table S12). For example, the group of species, *A. radiata* , *C. niger complex*, and *T. marginata*, share no statistically clear pairwise differences in average experienced habitat temperatures by the letter “d” above boxplots. Colors represent the gradient from low (blue) to high (orange) temperatures.

Average experienced habitat temperatures across the entire study season during “active” time differed among species (*p* value < 0.001) and log body mass (*p* value < 0.001), but not sex (*p* value = 0.12, Table 6). These results were confirmed when using data for the entire day (Table S11 and Figure S1). All species showed higher average experienced habitat temperatures during “active” time compared to all times during the day (data viz. Table 5). Among all the species, *T. carolina* (avg = 22.47°C) exhibited the lowest average experienced habitat temperature, while *A. radiata* and *C. niger* (avg = 28.64°C for both species) exhibited the highest (Figure 4, data viz. Table 3). *Terrapene carolina* significantly differed in average experienced habitat temperatures from all the other eight species (*p* values < 0.05, Figure 4, Table S12). *Chelonoidis denticulata* also differed from the other species (*p* values < 0.05), except for *T. graeca* (*p* value = 0.39, mean difference = 1.14°C) and *T. hermanni* (*p* value = 0.09, mean difference = 1.30°C). Finally, *A. radiata* differed from all other species (*p* values < 0.05) except for *C. niger* (*p* value = 1.0, mean difference = −0.005°C) and *T. marginata* (*p* value = 0.34, mean difference = 1.02°C) (Table S12). We obtained similar results when the analyses were run using the data from the entire day (Table S13).

**TABLE 6:** Influence of species, log body mass, and sex on experienced habitat temperatures (T_exp_) across (all 9 species) and within (only *C. carbonaria* , *C. denticulata* , *T. hermanni* , *T. marginata* , and *A. radiata* ) species.

Source	df	Res df	SS	RSS	MS	*F*	*p*
Avg *T*_exp_ (9 species)							
Species	8	64	198.20	62.68	24.77	25.3	**< 2.0 × 10** ^**−16**^
Log body mass	1	71	74.12	186.76	74.12	28.18	**1.21 × 10** ^**−6**^
Sex	1	71	8.81	252.07	8.07	2.48	0.12
Avg *T*_exp_ (5 species)							
Species	4	40	58.31	32.90	14.57	17.72	**1.91 × 10** ^**−8**^
Sex	1	40	10.32	32.90	10.32	12.55	**0.001**
Species:Sex	4	40	7.23	32.90	1.80	2.19	0.08
Avg *T*_exp_ (5 species)							
Species	4	40	65.70	33.73	16.42	19.47	**5.79 × 10** ^**−9**^
Log body mass	1	40	11.92	33.73	11.92	14.13	**5.44 × 10** ^**−4**^
Species:Log body mass	4	40	4.79	33.73	1.19	1.42	0.24

*Note:* Data only for “active” temperatures (6 am–9 pm) across each month for the entire season. *p* values in bold represent statistical significance (< 0.05).

Abbreviations: df, degrees of freedom; MS, mean squares; Res df, residual degrees of freedom; RSS, residual sum of squares; SS = sum of squares.

Across the five species with at least ten individuals sampled ( *C. carbonaria* , *C. denticulata* , *T. hermanni* , *T. marginata* , and *A. radiata* ), males had slightly higher “active” experienced habitat temperatures (avg = 26.68°C) compared to females (avg = 26.56°C) (*p* value = 0.001, Table 6). However, within each species, only *C. denticulata* showed a significant difference in experienced “active” average temperatures between sexes (*p* value = 0.01, mean difference = 2.47°C, 95% CI = (0.37, 4.56), with males (avg = 26.99°C)) having higher “active” experienced habitat temperatures than females (avg = 24.52°C). Across all nine species, those with overall larger log body mass tended to select higher “active” experienced habitat temperatures compared to species with smaller log body mass (*p* value < 0.001, Table 6), with the exception of *C. denticulata* (avg log body mass = 8.61, avg. experienced temp = 25.26°C), which exhibited higher body mass than other species, but experienced lower “active” temperatures.

### 3.5 Intraspecific Variation in Experienced Habitat Temperatures

Within each species, individuals showed different “active” experienced habitat temperatures (Data viz. Table 4), and intraspecific variation (SD) varied across species (*p* value < 0.001) and log body mass (*p* value = 0.04), but not sex (*p* value = 0.31) (Table 7). For the five species with ten individuals, SD for “active” experienced habitat temperatures significantly differed across species (*p* value < 0.001) but not sex (*p* value = 0.49) or log body mass (*p* value = 0.79) (Table 7). These results were confirmed when using data for the entire day (Table S14).

**TABLE 7:** Influence of species, sex, and log body mass on intraspecific variation (SD) in “active” experienced habitat temperatures (6 am–9 pm) (T_exp_) across (all 9 species) and within (only *C. carbonaria* , *C. denticulata* , *T. hermanni* , *T. marginata* , and *A. radiata* ) species for each month for the entire season.

Source	df	Res df	SS	RSS	MS	*F*	*p*
SD *T*_exp_ (9 species)							
Species	8	64	32.70	13.84	4.08	18.9	**3.31 × 10** ^**−14**^
Log body mass	1	71	2.48	44.06	2.479	3.99	**0.04**
Sex	1	71	0.67	45.87	0.66	1.03	0.31
SD *T*_exp_ (5 species)							
Species	4	40	11.82	7.42	2.95	15.92	**7.04 × 10** ^**−8**^
Sex	1	40	0.08	7.42	0.08	0.47	0.49
Species:Sex	4	40	0.53	7.42	0.13	0.72	0.58
SD *T*_exp_ (5 species)							
Species	4	40	6.95	7.84	1.73	8.86	**3.18 × 10** ^**−5**^
Log body mass	1	40	0.01	7.84	0.01	0.07	0.79
Species:Log body mass	4	40	0.19	7.84	0.04	0.24	0.91

*Note:* *p* values in bold represent statistical significance (< 0.05).

Abbreviations: df, degrees of freedom; MS, mean squares; Res df, residual degrees of freedom; RSS, residual sum of squares; SS, sum of squares.

**FIGURE 5:**
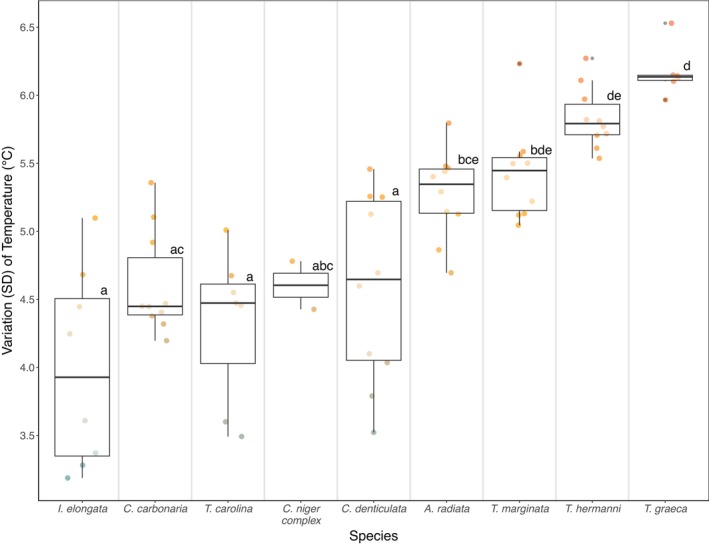
Variation (SD) of “active” experienced habitat temperatures across nine turtle species for each individual across the entire season. Letters shared above boxplots for each species denote no statistically clear differences in standard deviations of experienced “active” temperature. Letters not shared between boxplots for each species denote significant differences in standard deviations of experienced “active” temperature based on the Tukey post hoc test (Section S1 and Table S15). For example, the group of species, *T. graeca* , *T. hermanni* , and *T. marginata*, share no statistically clear pairwise differences in variation of experienced habitat temperatures by the letter “d” above boxplots. Colors represent the gradient from low (blue) to high (orange) variation in temperature.

Overall, *I. elongata* (avg SD = 3.99) and *T. graeca* (avg SD = 6.16) exhibited the lowest and highest variation (SD), respectively, of “active” experienced habitat temperatures (Figure 5). Species with larger body mass tended to have lower variation in experienced habitat temperatures during “active” time, whereas species with smaller body mass tended to have higher variation in experienced habitat temperatures during “active” time with the exception of *T. carolina* (avg log body mass = 6.13, avg. SD = 4.32) and *I. elongata* (avg log body mass = 7.69, avg. SD = 3.99), which have smaller log body mass but exhibited lower variation in experienced habitat temperatures during “active” time (see Figure S2 for species comparison for data across the entire day). Detailed results of Tukey post hoc tests comparing across and within species for both “active” time and during the entire day can be found in the Section S1, S2 and Tables S15–S18.

## 4 Discussion

We examined experienced habitat temperatures in nine turtle species that differ in native habitat, body size, and other ecological traits. Our results focus on thermal habitat use under semi‐natural conditions, not internal body temperatures or thermal performance limits. While internal body temperature is influenced by physiology, morphology, and inertia, the temperatures reported here reflect the thermal environments turtles used from those available to them during the study season at the study site. Across species, we found clear differences in average experienced habitat temperatures, both during active hours (sunrise to sunset) and over the full 24‐h cycle. In all species, experienced habitat temperatures were higher during active hours, likely due to a combination of warmer environmental conditions and thermoregulatory behaviors such as basking. Basking has been documented in five of the nine species in our study: *T. carolina* , *T. graeca* , *T. hermanni* , *T. marginata* , and *A. radiata* . (Boucher 1999; Meek and Jayes 1982; Willemsen and Hailey 2002; Fasola et al. 2002; Highfield 2018; Castellano et al. 2013). In addition to behavior, thermoregulation in turtles could be affected by morphological differences such as carapace coloration (Maki et al. 2025). However, the use of coloration for thermoregulation in ectotherms may be species‐specific (Smith et al. 2016; Gunderson et al. 2022; Thompson et al. 2023; but see Hastings et al. 2023). Although not quantified in this study, the relationship between carapace coloration and thermoregulation in different turtle species should be considered in the future, but specific experimental designs are needed (Mochales‐Riaño et al. 2024).

During active hours (sunrise to sunset), *C. carbonaria* , *T. marginata* , and *T. graeca* experienced habitat temperatures that fell within the intermediate range of conditions found in their native environments. This suggests that they may be able to behaviorally thermoregulate within existing microhabitats. Among them, *C. carbonaria* showed especially limited variation among individuals, indicating constrained thermal ranges centered around the midpoint of its native temperature range. In contrast, *T. hermanni* experienced habitat temperatures near the upper end of its native thermal range and differed significantly from shaded microclimates. This suggests that *T. hermanni* may be relatively resilient to moderate climate warming, as long as future temperatures do not exceed the upper limits of its current thermal range. By comparison, *T. carolina* , *I. elongata* , and *C. denticulata* experienced habitat temperatures near the lower end of their native ranges and differed significantly from native sun microclimates. These three species—especially *T. carolina* and *I. elongata* —also showed low individual variability, consistently selecting cooler temperatures. This narrow thermal range may make them particularly vulnerable to future warming and changes in vegetation structure that alter thermal availability. Finally, *A. radiata* and *C. niger* experienced habitat temperatures above those typically observed in their native habitats during the July–September period. Overall, our findings highlight that behavioral plasticity in thermal habitat use may be a key mechanism shaping how individuals interact with their thermal environment, with important implications for understanding resilience in changing or managed habitats.

Comparing species under shared captive conditions revealed that turtles from tropical climates generally experienced higher temperatures during the time in which they are active than temperate or Mediterranean species. An exception was *C. denticulata* , a tropical species whose experienced habitat temperatures resembled those of Mediterranean species like *T. graeca* and *T. hermanni* . This may reflect *C. denticulata* 's ecology—favoring humid, closed forests and showing peak activity during cooler, wetter periods (Moskovits 1988; Moreira 1989; Farias et al. 2007; Böhm 2011).

*Astrochelys radiata* and *C. niger* exhibited the highest average experienced habitat temperatures, consistent with their warmer native habitats and larger body sizes (Blake, Parlin, et al. 2021; Blake, Tapia, et al. 2021; Stevenson 1985; Peralta‐Maraver and Rezende 2021; Terespolsky and Brereton 2021; Durrell et al. 1989; Leuteritz and Ravolanaivo 2005). Body mass, which influences thermal inertia, may help explain these thermal differences: larger‐bodied species tend to retain heat longer and may tolerate higher temperatures, while smaller‐bodied species experience more rapid shifts in body temperature (Stevenson 1985; Bulté and Blouin‐Demers 2010). These relationships may also account for differences observed among Mediterranean species. *T. marginata* , which has a significantly greater body mass than *T. graeca* and *T. hermanni* , experienced higher temperatures during the hours at which they are active. In addition to body size, habitat preferences likely play a role: *T. graeca* and *T. hermanni* are typically found in grasslands and shrublands with patchy vegetation and available shade (Berardo et al. 2015; Rozylowicz and Popescu 2013; Anadón et al. 2006), whereas *T. marginata* occupies warmer, denser environments such as garrigue and olive groves (Sperone 2020).

*Terrapene carolina* , the only non‐tortoise species in our study, consistently experienced cooler habitat temperatures than all other species and showed low variation among individuals—despite its relatively small body mass and correspondingly lower thermal inertia. This suggests a reliance on thermally stable, shaded environments, such as deciduous forests, consistent with its wild behavior of being active during cooler, more humid periods, particularly in the early morning and evening (Fredericksen 2014; Dodd 2002).

To summarize, we found that variation in body mass among species generally correlates positively with the average experienced habitat temperatures. However, exceptions such as *C. denticulata* suggest that native microhabitat conditions and species‐specific ecology may influence experienced habitat temperatures more strongly than body mass. Intraspecific variation (SD) in experienced habitat temperatures largely follows this trend, being shaped by differences in body mass, native environment, or both. For instance, the lower intraspecific temperature variation observed in *T. carolina* likely reflects the narrow thermal tolerance of this species, whereas the greater variation seen in *T. hermanni* and *T. graeca* may result from individuals having smaller body masses and broader thermal ranges, consistent with the thermally variable environments of their native range. *Indotestudo elongata's* lower intraspecific variation of experienced habitat temperatures could be affected by large body mass, native microhabitat preference, and time of activity in the wild corresponding to crepuscular hours of the day in order to avoid high air temperatures (Rahman et al. 2019).

While sexual size dimorphism can influence thermoregulatory behavior (Berry and Shine 1980; Agha et al. 2018; Bulté and Blouin‐Demers 2010), we found that despite *T. hermanni* having females larger than males, both sexes experienced similar temperatures. On the other hand, in *C. denticulata* , despite the two sexes having similar body mass, males experienced higher average temperatures than females during “active” hours. For *C. denticulata* , our results corroborate previous work (Tavares et al. 2019) suggesting that factors other than body mass may influence thermal ranges in this species. In *T. hermanni* , different habitat use among sexes could also explain different experienced habitat temperatures. However, data supporting a different habitat use between males and females are mixed, with some studies reporting sex‐related differences in movement (Longepierre et al. 2001; Fasola et al. 2002), while others do not (Rozylowicz and Popescu 2013; Harris et al. 2020). These discrepancies may reflect the timing of observations and/or thermal availability, as behaviors such as mating or nesting can influence movement and habitat use (Dodd 2002; Longepierre et al. 2001).

An important consideration in this study is that thermoregulatory behavior could be influenced by the captive, semi‐natural environment of A Cupulatta. For example, artificial food resources, spatial availability, and reduced environmental complexity will likely be different from those in the native habitats of each species. However, without a comparative study involving multiple species within the same environment, we would be unable to determine the intrinsic factors shaping thermoregulatory divergence.

## 5 Conclusions

Our study demonstrates that body mass and variation in temperature in the native habitat influence experienced habitat temperatures across turtles, highlighting the role of biological and evolutionary factors in shaping thermoregulatory behavior across species. Facilities like A Cupulatta, which house diverse species under standardized conditions, offer a unique opportunity to compare species from different climatic backgrounds in a controlled setting. This allowed us to examine not only behavioral responses to temperatures typical of native habitats but also habitat use in response to sustained high temperatures, such as those that may occur during heatwaves. Our findings provide valuable data on species‐specific thermal differences and their dependence on particular microclimates, revealing potential vulnerabilities if these thermal refuges are lost in the wild. In particular, species that rely on cooler microhabitats for effective thermoregulation may be at heightened risk as ambient temperatures continue to rise. While temperatures recorded by the data loggers may partly reflect heat exchange between the tortoises and their immediate surroundings, this does not undermine our conclusions, as this effect is systematic across the recording period and comparable among species. Importantly, differences in body mass, which are expected to modulate heat transfer, are explicitly incorporated into our analyses.

In terms of management, understanding how body mass, sex, and microclimate shape thermal behavior is crucial for husbandry and conservation—particularly since over 130 threatened turtle species are currently housed in zoos worldwide (Ginal et al. 2023). Captive environments must account for these differences—both between individuals of different body mass and among species—when designing enclosures that support optimal thermoregulation. This is especially important as climate change continues to alter environmental conditions. Future conservation strategies should consider thermal ecology, habitat selection, and life history traits—such as body mass and sex—when assessing species sensitivity to environmental change.

## Author Contributions

**Emma White:** data curation (equal), formal analysis (lead), visualization (equal), writing – original draft (lead). **Jana Stupavsky:** data curation (equal), software (equal), visualization (lead), writing – review and editing (equal). **Brandon T. Hastings:** data curation (equal), writing – review and editing (equal). **Austin Ray:** data curation (equal). **Miguel A. Carretero:** conceptualization (supporting), investigation (equal), methodology (equal), resources (equal), writing – review and editing (equal). **Pierre Moisson:** investigation (equal), methodology (equal), resources (equal), writing – review and editing (equal). **Julien Claude:** formal analysis (equal), methodology (equal), supervision (equal), writing – review and editing (equal). **Scott Glaberman:** conceptualization (lead), data curation (equal), investigation (equal), methodology (equal), project administration (supporting), resources (equal), writing – original draft (supporting), writing – review and editing (equal). **Ylenia Chiari:** conceptualization (equal), data curation (equal), funding acquisition (lead), investigation (equal), methodology (equal), project administration (lead), resources (equal), software (lead), supervision (lead), visualization (supporting), writing – original draft (equal).

## Funding

This project was partially supported by funding from the Percy Sladen Memorial Fund and the British Chelonia Group. Some data‐loggers were provided by project PTDC/BIA‐BEC/101256/2008 funded by Fundação para a Ciência e a Tecnologia (Portugal).

## Conflicts of Interest

The authors declare no conflicts of interest.

## Supporting information


**Figure S1:** Average experienced habitat temperatures for each individual of the nine turtle species during the entire day across the season.
**Figure S2:** Variation (SD) of experienced habitat temperatures across nine turtle species for each individual for the entire day across the entire season.
**Table S1:** Influence of sex on log body mass within each species as obtained by running a two‐way ANOVA and Tukey post hoc test.
**Table S2:** Games‐Howell post hoc species comparisons for native sun microclimate temperatures during “active” time (6 am–9 pm) for months July–September.
**Table S3:** Games‐Howell post hoc species comparisons for native shade microclimate temperatures during “active” time (6 am–9 pm) for months July–September.
**Table S4:** Games‐Howell post hoc species comparisons for combined native shade and sun microclimate temperatures during “active” time (6 am–9 pm) for months July–September.
**Table S5:** Influence of species on native sun, shade, and combined shade and sun microclimate temperatures during all times of day for months July–September.
**Table S6:** Games‐Howell post hoc species comparisons for native sun microclimate temperatures during all times of the day for months July–September.
**Table S7:** Games‐Howell post hoc species comparisons for native shade microclimate temperatures during all times of the day for months July–September.
**Table S8:** Games‐Howell post hoc species comparisons for combined native shade and sun microclimate temperatures during all times of the day for months July–September.
**Table S9:** Two‐sample Wilcoxon rank sum tests comparisons of experienced habitat versus native microclimate temperatures (sun, shade, and sun and shade combined) during all times of day by species.
**Table S10:** Two‐sample *t*‐test comparisons of experienced habitat temperatures and enclosure temperatures (sun or shade) during all times of day for each species.
**Table S11:** Influence of species, log body mass, and sex on experienced habitat temperatures (T_exp_) across (all 9 species) and within species (only *C. carbonaria* , *C. denticulata* , *T. hermanni* , *T. marginata* , and *A. radiata* ).
**Table S12:** Tukey post hoc pairwise species comparisons for “active” experienced habitat temperatures (6 am–9 pm) across each month for the entire season.
**Table S13:** Tukey post hoc pairwise species comparisons for experienced habitat temperatures for the entire day across all months for the entire season.
**Table S14:** Influence of species, log body mass, and sex on intraspecific variation (SD) in experienced habitat temperatures (*T*_exp_) across (all 9 species) and within species (only *C. carbonaria* , *C. denticulata* , *T. hermanni* , *T. marginata* , and *A. radiata* ).
**Table S15:** Tukey post hoc pairwise species comparisons for variation (SD) in “active” experienced habitat temperatures (6 am–9 pm) across each month for the entire season.
**Table S16:** Tukey post hoc pairwise species comparisons for variation (SD) in experienced habitat temperatures for the entire day across all months for the entire season.
**Table S17:** Influence of sex on variation (SD) in experienced habitat temperatures within species. Data for “active” temperatures (6am to 9pm).
**Table S18:** Influence of sex on variation (SD) in experienced habitat temperatures within species. Data for all times during the day.

## Data Availability

The entire dataset and the R codes used for the analyses can be found on Dryad https://doi.org/10.5061/dryad.5x69p8dhg. Code for filtering turtle experienced habitat temperature data can be found here: https://github.com/brandon‐hastings/thermal_data_cleaning.
