# Supplementary material for: Drivers of Thermal Habitat Use in Turtles Studied Under Semi‐Natural Conditions
Source: Ecol Evol. 2026 Apr 7;16(4):e73325. doi: 10.1002/ece3.73325 (PMC13054840; doi:10.1002/ece3.73325)
Supplement: Supplementary file 1 — Figure S1: Average experienced habitat temperatures for each individual of the nine turtle species during the entire day across the season. Figure S2: Variation (SD) of experienced habitat temperatures across nine turtle species for each individual for the entire day across the entire season. Table S1: Influence of sex on log body mass within each species as obtained by running a two‐way ANOVA and Tukey post hoc test. Table S2: Games‐Howell post hoc species comparisons for native sun microclimate temperatures during “active” time (6 am–9 pm) for months July–September. Table S3: Games‐Howell post hoc species comparisons for native shade microclimate temperatures during “active” time (6 am–9 pm) for months July–September. Table S4: Games‐Howell post hoc species comparisons for combined native shade and sun microclimate temperatures during “active” time (6 am–9 pm) for months July–September. Table S5: Influence of species on native sun, shade, and combined shade and sun microclimate temperatures during all times of day for months July–September. Table S6: Games‐Howell post hoc species comparisons for native sun microclimate temperatures during all times of the day for months July–September. Table S7: Games‐Howell post hoc species comparisons for native shade microclimate temperatures during all times of the day for months July–September. Table S8: Games‐Howell post hoc species comparisons for combined native shade and sun microclimate temperatures during all times of the day for months July–September. Table S9: Two‐sample Wilcoxon rank sum tests comparisons of experienced habitat versus native microclimate temperatures (sun, shade, and sun and shade combined) during all times of day by species. Table S10: Two‐sample t‐test comparisons of experienced habitat temperatures and enclosure temperatures (sun or shade) during all times of day for each species. Table S11: Influence of species, log body mass, and sex on experienced habitat temperatures (Texp) across (all 9 speci [file ECE3-16-e73325-s001.docx]

**SUPPLEMENTARY MATERIALS**

**Table S1:** Influence of sex on log body mass within each species as obtained by running a two-way ANOVA and Tukey post-hoc test. *C. niger* complex is not included in this analysis, as data on sexual mass dimorphism is provided in Chiari, 2020 and only two individuals are included in this study. *T. graeca* is not included as only males were used in this study. P-values in bold represent statistical significance (<0.05) (CI = confidence interval), Mean difference in log body mass = male log body mass avg – female log body mass avg).

| Species | Average Body Mass for Males (g) | Average Body Mass for Females (g) | Mean difference in log body mass (g) | 95% CI (Lower, Upper) | p-value |
| --- | --- | --- | --- | --- | --- |
| *C. carbonaria* | 4656.6 | 4927.1 | 0.04 | (-0.75, 0.85) | 1.00 |
| *T. carolina* | 437.6 | 507.2 | -0.11 | (-1.00, 0.77) | 1.00 |
| *C. denticulata* | 5836.6 | 6324.2 | -0.02 | (-0.83, 0.77) | 1.00 |
| *I. elongata* | 2023.5 | 2473.7 | -0.22 | (-1.04, 0.60) | 0.99 |
| *T. hermanni* | 497.0 | 785.6 | -0.47 | (-1.21, 0.26) | 0.65 |
| *A. radiata* | 8804.0 | 7896.0 | 0.11 | (-0.62, 0.85) | 1.00 |
| *T. marginata* | 3979.6 | 2860.4 | 0.36 | (-0.43, 1.17) | 0.96 |

**Table S2:** Games-Howell post-hoc species comparisons for native sun microclimate temperatures during “active” time (6 am to 9 pm) for months July-September. P-values in bold represent statistical significance (<0.05).

| Species 1 | Species 2 | Mean difference (^o^C) | 95% CI (Lower, Upper) | SE (^o^C) | t-value | df | p-value |
| --- | --- | --- | --- | --- | --- | --- | --- |
| *C. carbonaria* | *T. carolina* | -3.37 | (-3.67, -3.07) | 0.06 | 34.7 | 637 | **0** |
|  | *C. denticulata* | -0.70 | (-1.18, -0.22) | 0.10 | 4.58 | 947 | **1.83 x 10^-4^** |
|  | *I. elongata* | -0.17 | (-0.68, 0.34) | 0.11 | 1.04 | 272 | 0.98 |
|  | *C. niger complex* | -5.58 | (-5.96, -5.20) | 0.08 | 45.9 | 672 | **0** |
|  | *T. graeca* | 1.11 | (0.77, 1.45) | 0.07 | 10.2 | 982 | **4.91 x 10^-13^** |
|  | *T. hermanni* | -1.13 | (-1.44, -0.82) | 0.07 | 11.4 | 696 | **0** |
|  | *T. marginata* | 1.33 | (0.86, 1.79) | 0.10 | 8.99 | 909 | **0** |
|  | *A. radiata* | -3.44 | (-4.20, -2.68) | 0.16 | 14.4 | 115 | **5.21 x 10^-14^** |
| *T. carolina* | *C. denticulata* | 2.66 | (2.29, 3.04) | 0.08 | 22.1 | 467 | **1.53 x 10^-11^** |
|  | *I. elongata* | 3.20 | (2.78, 3.62) | 0.09 | 23.9 | 126 | **0** |
|  | *C. niger complex* | -2.21 | (-2.44, -1.97) | 0.05 | 29.5 | 171 | **0** |
|  | *T. graeca* | 4.48 | (4.32, 4.64) | 0.03 | 87.1 | 3786 | **1.36 x 10^-8^** |
|  | *T. hermanni* | 2.24 | (2.16, 2.32) | 0.01 | 83.9 | 9233 | **0** |
|  | *T. marginata* | 4.70 | (4.35, 5.05) | 0.07 | 41.7 | 418 | **0** |
|  | *A. radiata* | -0.07 | (-0.77, 0.62) | 0.15 | 0.33 | 81.5 | 1.0 |
| *C. denticulata* | *I. elongata* | 0.53 | (-0.02, 1.10) | 0.12 | 2.98 | 345 | 0.07 |
|  | *C. niger complex* | -4.87 | (-5.31, -4.43) | 0.09 | 34.5 | 619 | **2.47 x 10^-10^** |
|  | *T. graeca* | 1.81 | (1.41, 2.22) | 0.09 | 13.9 | 628 | **1.06 x 10^-10^** |
|  | *T. hermanni* | -0.42 | (-0.80, -0.04) | 0.08 | 3.47 | 495 | **0.01** |
|  | *T. marginata* | 2.03 | (1.52, 2.54) | 0.11 | 12.4 | 867 | **0** |
|  | *A. radiata* | -2.74 | (-3.53, -1.95) | 0.17 | 10.9 | 135 | **0** |
| *I. elongata* | *C. niger complex* | -5.41 | (-5.89, -4.93) | 0.10 | 35.4 | 197 | **0** |
|  | *T. graeca* | 1.28 | (0.83, 1.73) | 0.10 | 8.97 | 161 | **9.74 x 10^-14^** |
|  | *T. hermanni* | -0.96 | (-1.39, -0.53) | 0.09 | 7.08 | 132 | **2.83 x 10^-9^** |
|  | *T. marginata* | 1.50 | (0.95, 2.04) | 0.12 | 8.60 | 313 | **1.10 x 10^-12^** |
|  | *A. radiata* | -3.27 | (-4.08, -2.46) | 0.18 | 12.7 | 140 | **0** |
| *C. niger complex* | *T. graeca* | 6.69 | (6.41, 6.96) | 0.06 | 74.9 | 341 | **0** |
|  | *T. hermanni* | 4.45 | (4.20, 4.69) | 0.05 | 57.2 | 198 | **0** |
|  | *T. marginata* | 6.91 | (6.49, 7.32) | 0.09 | 51.5 | 570 | **4.94 x 10^-10^** |
|  | *A. radiata* | 2.13 | (1.40, 2.87) | 0.16 | 9.22 | 99.8 | **1.45 x 10^-10^** |
| *T. graeca* | *T. hermanni* | -2.24 | (-2.41, -2.07) | 0.03 | 40.4 | 4972 | **0** |
|  | *T. marginata* | 0.220 | (-0.16, 0.60) | 0.08 | 1.79 | 586 | 0.68 |
|  | *A. radiata* | -4.55 | (-5.27, -3.84) | 0.15 | 20.2 | 89.6 | **4.23 x 10^-10^** |
| *T. hermanni* | *T. marginata* | 2.46 | (2.10, 2.82) | 0.08 | 21.5 | 447 | **0** |
|  | *A. radiata* | -2.31 | (-3.01, -1.61) | 0.15 | 10.5 | 82.9 | **1.07 x 10^-10^** |
| *T. marginata* | *A. radiata* | -4.77 | (-5.55, -3.99) | 0.17 | 19.4 | 127 | **0** |

**Table S3:** Games-Howell post-hoc species comparisons for native shade microclimate temperatures during “active” time (6 am to 9 pm) for months July-September. P-values in bold represent statistical significance (<0.05).

| Species 1 | Species 2 | Mean difference (^o^C) | 95% CI (Lower, Upper) | SE (^o^C) | t-value | df | p-value |
| --- | --- | --- | --- | --- | --- | --- | --- |
| *C. carbonaria* | *T. carolina* | -3.52 | (-3.82, -3.22) | 0.06 | 36.2 | 635 | **0** |
|  | *C. denticulata* | -0.69 | (-1.14, -0.25) | 0.10 | 4.86 | 1018 | **4.85 x 10^-5^** |
|  | *I. elongata* | 1.45 | (0.95, 1.95) | 0.11 | 9.09 | 289 | **8.95 x 10^-13^** |
|  | *C. niger complex* | -3.02 | (-3.40, -2.64) | 0.08 | 24.5 | 651 | **0** |
|  | *T. graeca* | -0.83 | (-1.16, -0.51) | 0.07 | 8.09 | 809 | **1.56 x 10^-13^** |
|  | *T. hermanni* | -2.13 | (-2.44, -1.82) | 0.07 | 21.4 | 697 | **0** |
|  | *T. marginata* | -0.60 | (-0.99, -0.21) | 0.08 | 4.85 | 1027 | **5.0 x 10^-5^** |
|  | *A. radiata* | -2.78 | (-3.49, -2.07) | 0.15 | 12.4 | 121 | **1.07 x 10^-13^** |
| *T. carolina* | *C. denticulata* | 2.83 | (2.50, 3.16) | 0.07 | 26.7 | 469 | **1.92 x 10^-11^** |
|  | *I. elongata* | 4.97 | (4.57, 5.37) | 0.09 | 39.0 | 126 | **0** |
|  | *C. niger complex* | 0.50 | (0.25, 0.74) | 0.05 | 6.45 | 169 | **4.0 x 10^-8^** |
|  | *T. graeca* | 2.69 | (2.57, 2.81) | 0.02 | 69.6 | 4050 | **2.8 x 10^-8^** |
|  | *T. hermanni* | 1.39 | (1.31, 1.47) | 0.01 | 52.3 | 8834 | **0** |
|  | *T. marginata* | 2.92 | (2.67, 3.16) | 0.05 | 36.6 | 426 | **0** |
|  | *A. radiata* | 0.74 | (0.09, 1.39) | 0.14 | 3.65 | 81.5 | **0.01** |
| *C. denticulata* | *I. elongata* | 2.14 | (1.63, 2.66) | 0.11 | 13.0 | 313 | **1.19 x 10^-12^** |
|  | *C. niger complex* | -2.33 | (-2.73, -1.92) | 0.09 | 17.9 | 597 | **4.65 x 10^-10^** |
|  | *T. graeca* | -0.14 | (-0.48, 0.20) | 0.07 | 1.28 | 576 | 0.93 |
|  | *T. hermanni* | -1.44 | (-1.77, -1.10) | 0.07 | 13.3 | 507 | **1.71 x 10^-10^** |
|  | *T. marginata* | 0.08 | (-0.32, 0.49) | 0.09 | 0.67 | 825 | 0.99 |
|  | *A. radiata* | -2.08 | (-2.81, -1.36) | 0.16 | 9.11 | 129 | **9.02 x 10^-14^** |
| *I. elongata* | *C. niger complex* | -4.47 | (-4.93, -4.00) | 0.10 | 30.1 | 210 | **1.23 x 10^-14^** |
|  | *T. graeca* | -2.28 | (-2.70, -1.87) | 0.09 | 17.3 | 146 | **2.38 x 10^-14^** |
|  | *T. hermanni* | -3.58 | (-3.99, -3.17) | 0.09 | 27.7 | 133 | **0** |
|  | *T. marginata* | -2.05 | (-2.52, -1.59) | 0.10 | 13.7 | 228 | **4.54 x 10^-14^** |
|  | *A. radiata* | -4.23 | (-4.98, -3.47) | 0.16 | 17.6 | 142 | **0** |
| *C. niger complex* | *T. graeca* | 2.18 | (1.92, 2.45) | 0.06 | 25.6 | 245 | **1.05 x 10^-13^** |
|  | *T. hermanni* | 0.89 | (0.63, 1.14) | 0.05 | 11.1 | 196 | **0** |
|  | *T. marginata* | 2.42 | (2.07, 2.76) | 0.07 | 21.9 | 475 | **3.54 x 10^-11^** |
|  | *A. radiata* | 0.24 | (-0.44, 0.93) | 0.15 | 1.12 | 105 | 0.97 |
| *T. graeca* | *T. hermanni* | -1.29 | (-1.43, -1.16) | 0.03 | 29.3 | 6276 | **0** |
|  | *T. marginata* | 0.23 | (-0.03, 0.50) | 0.06 | 2.65 | 604 | 0.16 |
|  | *A. radiata* | -1.94 | (-2.60, -1.28) | 0.14 | 9.41 | 86.4 | **2.81 x 10^-10^** |
| *T. hermanni* | *T. marginata* | 1.52 | (1.27, 1.78) | 0.05 | 18.5 | 489 | **8.40 x 10^-11^** |
|  | *A. radiata* | -0.64 | (-1.30, 0.002) | 0.14 | 3.17 | 83.3 | 0.05 |
| *T. marginata* | *A. radiata* | -2.17 | (-2.86, -1.48) | 0.15 | 9.97 | 107 | **1.08 x 10^-13^** |

**Table S4:** Games-Howell post-hoc species comparisons for combined native shade and sun microclimate temperatures during “active” time (6 am to 9 pm) for months July-September. P-values in bold represent statistical significance (<0.05).

| Species 1 | Species 2 | Mean difference (^o^C) | 95% CI (Lower, Upper) | SE (^o^C) | t-value | df | p-value |
| --- | --- | --- | --- | --- | --- | --- | --- |
| *C. carbonaria* | *T. carolina* | -3.45 | (-3.74, -3.15) | 0.06 | 36.6 | 636 | **0** |
|  | *C. denticulata* | -0.69 | (-1.15, -0.24) | 0.10 | 4.82 | 973 | **5.87 x 10^-5^** |
|  | *I. elongata* | 0.63 | (0.14, 1.13) | 0.11 | 4.06 | 278 | **2.0 x 10^-3^** |
|  | *C. niger complex* | -4.30 | (-4.67, -3.93) | 0.08 | 35.9 | 647 | **0** |
|  | *T. graeca* | 0.13 | (-0.18, 0.45) | 0.07 | 1.33 | 882 | 0.92 |
|  | *T. hermanni* | 0.45 | (0.15, 0.75) | 0.06 | 4.76 | 703 | **8.27 x 10^-5^** |
|  | *T. marginata* | 0.36 | (-0.04, 0.76) | 0.09 | 2.78 | 995 | 0.12 |
|  | *A. radiata* | -3.11 | (-3.83, -2.39) | 0.16 | 13.7 | 116 | **2.34 x 10^-14^** |
| *T. carolina* | *C. denticulata* | 2.75 | (2.40, 3.09) | 0.07 | 24.6 | 467 | **1.64 x 10^-11^** |
|  | *I. elongata* | 4.08 | (3.68, 4.49) | 0.09 | 32.1 | 126 | **0** |
|  | *C. niger complex* | -0.85 | (-1.09, -0.61) | 0.05 | 11.3 | 169 | **7.26 x 10^-14^** |
|  | *T. graeca* | 3.58 | (3.45, 3.72) | 0.03 | 83.0 | 3905 | **1.98 x 10^-8^** |
|  | *T. hermanni* | 3.90 | (3.82, 3.99) | 0.01 | 147 | 8836 | **8.37 x 10^-12^** |
|  | *T. marginata* | 3.81 | (3.52, 4.09) | 0.06 | 41.6 | 422 | **0** |
|  | *A. radiata* | 0.33 | (-0.32, 0.99) | 0.14 | 1.61 | 81.5 | 0.79 |
| *C. denticulata* | *I. elongata* | 1.34 | (0.81, 1.87) | 0.11 | 7.94 | 334 | **1.59 x 10^-12^** |
|  | *C. niger complex* | -3.60 | (-4.02, -3.18) | 0.09 | 26.9 | 612 | **3.45 x 10^-10^** |
|  | *T. graeca* | 0.83 | (0.46, 1.21) | 0.08 | 7.04 | 594 | **6.69 x 10^-10^** |
|  | *T. hermanni* | 1.16 | (0.80, 1.51) | 0.08 | 10.2 | 502 | **1.44 x 10^-10^** |
|  | *T. marginata* | 1.06 | (0.61, 1.51) | 0.10 | 7.40 | 850 | **1.23 x 10^-11^** |
|  | *A. radiata* | -2.41 | (-3.15, -1.67) | 0.16 | 10.2 | 132 | **8.65 x 10^-14^** |
| *I. elongata* | *C. niger complex* | -4.94 | (-5.40, -4.48) | 0.10 | 33.5 | 206 | **0** |
|  | *T. graeca* | -0.50 | (-0.92, -0.08) | 0.09 | 3.7 | 152 | **7.0 x 10^-3^** |
|  | *T. hermanni* | -0.18 | (-0.58, 0.22) | 0.09 | 1.40 | 133 | 0.89 |
|  | *T. marginata* | -0.27 | (-0.76, 0.21) | 0.11 | 1.78 | 263 | 0.69 |
|  | *A. radiata* | -3.75 | (-4.52, -2.98) | 0.17 | 15.4 | 140 | **0** |
| *C. niger complex* | *T. graeca* | 4.43 | (4.17, 4.70) | 0.06 | 51.8 | 277 | **2.31 x 10^-13^** |
|  | *T. hermanni* | 4.76 | (4.51, 5.00) | 0.05 | 60.5 | 197 | **0** |
|  | *T. marginata* | 4.66 | (4.29, 5.03) | 0.08 | 39.6 | 533 | **3.16 x 10^-10^** |
|  | *A. radiata* | 1.19 | (0.48, 1.89) | 0.15 | 5.38 | 103 | **1.64 x 10^-5^** |
| *T. graeca* | *T. hermanni* | 0.32 | (0.17, 0.47) | 0.03 | 6.70 | 5693 | **8.10 x 10^-10^** |
|  | *T. marginata* | 0.22 | (-0.08, 0.53) | 0.07 | 2.26 | 596 | 0.36 |
|  | *A. radiata* | -3.25 | (-3.92, -2.57) | 0.15 | 15.3 | 87.6 | **3.39 x 10^-10^** |
| *T. hermanni* | *T. marginata* | -0.09 | (-0.39, 0.19) | 0.06 | 1.03 | 469 | 0.98 |
|  | *A. radiata* | -3.57 | (-4.24, -2.90) | 0.14 | 17.1 | 83.2 | **1.19 x 10^-10^** |
| *T. marginata* | *A. radiata* | -3.47 | (-4.19, -2.75) | 0.16 | 15.3 | 114 | **3.18 x 10^-14^** |

**Table S5:** Influence of species on native sun, shade, and combined shade and sun microclimate temperatures during all times of day for months July-September. P-values in bold represent statistical significance (<0.05).

| Source | dfn | dfd | F-value | p-value |
| --- | --- | --- | --- | --- |
| Native Sun Temp  Species | 8 | 815.63 | 628.05 | **< 2.2 x 10^-16^** |
| Native Shade Temp  Species | 8 | 815.33 | 519.91 | **< 2.2 x 10^-16^** |
| Native Sun & Shade Temp  Species | 8 | 815.24 | 613.5 | **< 2.2 x 10^-16^** |

**Table S6:** Games-Howell post-hoc species comparisons for native sun microclimate temperatures during all times of the day for months July-September. P-values in bold represent statistical significance (<0.05).

| Species 1 | Species 2 | Mean difference (^o^C) | 95% CI (Lower, Upper) | SE (^o^C) | t-value | df | p-value |
| --- | --- | --- | --- | --- | --- | --- | --- |
| *C. carbonaria* | *T. carolina* | -2.95 | (-3.25, -2.65) | 0.06 | 30.7 | 637 | **0** |
|  | *C. denticulata* | -0.68 | (-1.14, -0.22) | 0.10 | 4.63 | 982 | **1.41 x 10^-4^** |
|  | *I. elongata* | 1.00 | (0.50, 1.50) | 0.11 | 6.25 | 281 | **5.33 x 10^-8^** |
|  | *C. niger complex* | -4.68 | (-5.06, -4.30) | 0.08 | 38.7 | 661 | **0** |
|  | *T. graeca* | -1.02 | (-1.34, -0.69) | 0.07 | 9.68 | 904 | **0** |
|  | *T. hermanni* | -2.83 | (-3.13, -2.52) | 0.06 | 28.8 | 696 | **0** |
|  | *T. marginata* | -1.04 | (-1.43, -0.64) | 0.09 | 8.15 | 1020 | **3.19 x 10^-13^** |
|  | *A. radiata* | -4.88 | (-5.60, -4.17) | 0.15 | 21.7 | 119 | **2.09 x 10^-14^** |
| *T. carolina* | *C. denticulata* | 2.27 | (1.92, 2.62) | 0.07 | 20.2 | 468 | **1.88 x 10^-11^** |
|  | *I. elongata* | 3.95 | (3.55, 4.36) | 0.09 | 30.6 | 126 | **0** |
|  | *C. niger complex* | -1.73 | (-1.97, -1.49) | 0.05 | 22.9 | 170 | **6.22 x 10^-15^** |
|  | *T. graeca* | 1.94 | (1.79, 2.08) | 0.03 | 42.3 | 3900 | **1.96 x 10^-8^** |
|  | *T. hermanni* | 0.12 | (0.04, 0.2) | 0.01 | 4.71 | 9303 | **8.62 x 10^-5^** |
|  | *T. marginata* | 1.91 | (1.65, 2.18) | 0.06 | 22.5 | 425 | **0** |
|  | *A. radiata* | -1.93 | (-2.58, -1.28) | 0.14 | 9.44 | 81.6 | **4.70 x 10^-11^** |
| *C. denticulata* | *I. elongata* | 1.68 | (1.15, 2.21) | 0.12 | 9.87 | 331 | **7.22 x 10^-13^** |
|  | *C. niger complex* | -4.00 | (-4.42, -3.58) | 0.09 | 29.8 | 613 | **3.33 x 10^-10^** |
|  | *T. graeca* | -0.33 | (-0.71, 0.03) | 0.08 | 2.80 | 610 | 0.11 |
|  | *T. hermanni* | -2.15 | (-2.50, -1.79) | 0.08 | 18.8 | 500 | **1.32 x 10^-10^** |
|  | *T. marginata* | -0.35 | (-0.79, 0.07) | 0.09 | 2.55 | 828 | 0.21 |
|  | *A. radiata* | -4.20 | (-4.94, -3.47) | 0.16 | 18.1 | 135 | **0** |
| *I. elongata* | *C. niger complex* | -5.68 | (-6.15, -5.22) | 0.10 | 38.2 | 204 | **0** |
|  | *T. graeca* | -2.02 | (-2.45, -1.59) | 0.09 | 14.8 | 155 | **4.23 x 10^-14^** |
|  | *T. hermanni* | -3.83 | (-4.24, -3.42) | 0.09 | 29.3 | 132 | **0** |
|  | *T. marginata* | -2.04 | (-2.52, -1.56) | 0.10 | 13.2 | 240 | **0** |
|  | *A. radiata* | -5.88 | (-6.65, -5.12) | 0.17 | 24.4 | 143 | **0** |
| *C. niger complex* | *T. graeca* | 3.66 | (3.39, 3.94) | 0.06 | 42.2 | 293 | **9.91 x 10^-13^** |
|  | *T. hermanni* | 1.85 | (1.61, 2.10) | 0.05 | 23.7 | 196 | **5.22 x 10^-14^** |
|  | *T. marginata* | 3.64 | (3.29, 4.00) | 0.07 | 32.3 | 510 | **1.87 x 10^-10^** |
|  | *A. radiata* | -0.20 | (-0.89, 0.48) | 0.15 | 0.92 | 103 | 0.99 |
| *T. graeca* | *T. hermanni* | -1.81 | (-1.97, -1.66) | 0.03 | 36.2 | 5368 | **0** |
|  | *T. marginata* | -0.02 | (-0.31, 0.27) | 0.06 | 0.21 | 658 | 1.0 |
|  | *A. radiata* | -3.87 | (-4.53, -3.20) | 0.14 | 18.5 | 88.7 | **3.88 x 10^-10^** |
| *T. hermanni* | *T. marginata* | 1.79 | (1.52, 2.06) | 0.06 | 20.4 | 475 | **3.77 x 10^-11^** |
|  | *A. radiata* | -2.06 | (-2.71, -1.40) | 0.14 | 10.0 | 83.2 | **1.19 x 10^-10^** |
| *T. marginata* | *A. radiata* | -3.85 | (-4.54, -3.15) | 0.15 | 17.4 | 110 | **2.11 x 10^-15^** |

**Table S7:** Games-Howell post-hoc species comparisons for native shade microclimate temperatures during all times of the day for months July-September. P-values in bold represent statistical significance (<0.05).

| Species 1 | Species 2 | Mean difference (^o^C) | 95% CI (Lower, Upper) | SE (^o^C) | t-value | df | p-value |
| --- | --- | --- | --- | --- | --- | --- | --- |
| *C. carbonaria* | *T. carolina* | -3.26 | (-3.57, -2.95) | 0.06 | 33.2 | 635 | **0** |
|  | *C. denticulata* | -0.67 | (-1.11, -0.23) | 0.09 | 4.76 | 1036 | **7.73 x 10^-5^** |
|  | *I. elongata* | 1.49 | (0.99, 1.98) | 0.11 | 9.46 | 304 | **1.10 x 10^-12^** |
|  | *C. niger complex* | -2.85 | (-3.23, -2.46) | 0.08 | 22.9 | 660 | **0** |
|  | *T. graeca* | -2.08 | (-2.40, -1.75) | 0.07 | 19.9 | 809 | **0** |
|  | *T. hermanni* | -3.16 | (-3.47, -2.84) | 0.07 | 31.3 | 700 | **0** |
|  | *T. marginata* | -1.99 | (-2.37, -1.62) | 0.08 | 16.6 | 1013 | **3.56 x 10^-13^** |
|  | *A. radiata* | -4.19 | (-4.90, -3.48) | 0.15 | 18.7 | 122 | **2.24 x 10^-14^** |
| *T. carolina* | *C. denticulata* | 2.59 | (2.27, 2.91) | 0.07 | 25.2 | 469 | **2.04 x 10^-11^** |
|  | *I. elongata* | 4.75 | (4.36, 5.14) | 0.08 | 38.4 | 126 | **0** |
|  | *C. niger complex* | 0.41 | (0.17, 0.65) | 0.05 | 5.35 | 169 | **9.8 x 10^-6^** |
|  | *T. graeca* | 1.18 | (1.06, 1.30) | 0.02 | 30.3 | 4032 | **2.70 x 10^-8^** |
|  | *T. hermanni* | 0.10 | (0.01, 0.18) | 0.01 | 3.83 | 8667 | **4.0 x 10^-3^** |
|  | *T. marginata* | 1.27 | (1.04, 1.49) | 0.05 | 17.8 | 430 | **0** |
|  | *A. radiata* | -0.93 | (-1.58, -0.28) | 0.14 | 4.61 | 81.5 | **4.82 x 10^-4^** |
| *C. denticulata* | *I. elongata* | 2.16 | (1.66, 2.66) | 0.11 | 13.5 | 313 | **9.8 x 10^-13^** |
|  | *C. niger complex* | -2.17 | (-2.57, -1.78) | 0.09 | 17.0 | 591 | **4.91 x 10^-10^** |
|  | *T. graeca* | -1.41 | (-1.75, -1.07) | 0.07 | 12.9 | 587 | **5.0 x 10^-10^** |
|  | *T. hermanni* | -2.48 | (-2.81, -2.16) | 0.07 | 23.6 | 513 | **2.04 x 10^-10^** |
|  | *T. marginata* | -1.32 | (-1.71, -0.93) | 0.08 | 10.7 | 794 | **0** |
|  | *A. radiata* | -3.52 | (-4.24, -2.81) | 0.16 | 15.5 | 126 | **0** |
| *I. elongata* | *C. niger complex* | -4.33 | (-4.79, -3.88) | 0.10 | 29.9 | 214 | **0** |
|  | *T. graeca* | -3.57 | (-3.97, -3.16) | 0.09 | 27.7 | 148 | **4.02 x 10^-14^** |
|  | *T. hermanni* | -4.64 | (-5.04, -4.25) | 0.08 | 37.0 | 134 | **0** |
|  | *T. marginata* | -3.48 | (-3.93, -3.04) | 0.10 | 24.6 | 211 | **0** |
|  | *A. radiata* | -5.68 | (-6.43, -4.93) | 0.16 | 24.0 | 140 | **1.57 x 10^-14^** |
| *C. niger complex* | *T. graeca* | 0.76 | (0.50, 1.03) | 0.06 | 9.00 | 248 | **1.65 x 10^-13^** |
|  | *T. hermanni* | -0.31 | (-0.56, -0.05) | 0.05 | 3.85 | 198 | **5.0 x 10^-3^** |
|  | *T. marginata* | 0.85 | (0.52, 1.18) | 0.07 | 8.21 | 428 | **0** |
|  | *A. radiata* | -1.35 | (-2.03, -0.66) | 0.15 | 6.23 | 105 | **3.46 x 10^-7^** |
| *T. graeca* | *T. hermanni* | -1.08 | (-1.22, -0.93) | 0.03 | 24.0 | 6360 | **0** |
|  | *T. marginata* | 0.08 | (-0.16, 0.33) | 0.05 | 1.07 | 667 | 0.97 |
|  | *A. radiata* | -2.11 | (-2.77, -1.46) | 0.14 | 10.3 | 86.6 | **2.90 x 10^-10^** |
| *T. hermanni* | *T. marginata* | 1.16 | (0.93, 1.39) | 0.05 | 15.6 | 517 | **2.27 x 10^-10^** |
|  | *A. radiata* | -1.04 | (-1.69, -0.38) | 0.14 | 5.10 | 83.5 | **7.24 x 10^-5^** |
| *T. marginata* | *A. radiata* | -2.20 | (-2.88, -1.52) | 0.15 | 10.3 | 101 | **7.06 x 10^-14^** |

**Table S8:** Games-Howell post-hoc species comparisons for combined native shade and sun microclimate temperatures during all times of the day for months July-September. P-values in bold represent statistical significance (<0.05).

| Species 1 | Species 2 | Mean difference (^o^C) | 95% CI (Lower, Upper) | SE (^o^C) | t-value | df | p-value |
| --- | --- | --- | --- | --- | --- | --- | --- |
| *C. carbonaria* | *T. carolina* | -3.93 | (-4.25, -3.61) | 0.07 | 37.8 | 633 | **2.73 x 10^-12^** |
|  | *C. denticulata* | -1.50 | (-1.96, -1.04) | 0.10 | 10.1 | 1044 | **3.97 x 10^-13^** |
|  | *I. elongata* | 0.42 | (-0.07, 0.92) | 0.11 | 2.63 | 330 | 0.17 |
|  | *C. niger complex* | -4.59 | (-4.98, -4.19) | 0.09 | 35.9 | 698 | **0** |
|  | *T. graeca* | -2.37 | (-2.72, -2.03) | 0.07 | 21.4 | 812 | **8.59 x 10^-14^** |
|  | *T. hermanni* | -3.81 | (-4.14, -3.48) | 0.07 | 36.0 | 685 | **0** |
|  | *T. marginata* | -2.34 | (-2.73, -1.94) | 0.08 | 18.4 | 1013 | **2.01 x 10^-13^** |
|  | *A. radiata* | -5.36 | (-6.08, -4.65) | 0.16 | 23.7 | 128 | **0** |
| *T. carolina* | *C. denticulata* | 2.43 | (2.10, 2.76) | 0.07 | 22.8 | 469 | **1.88 x 10^-11^** |
|  | *I. elongata* | 4.35 | (3.96, 4.74) | 0.08 | 35.4 | 126 | **1.89 x 10^-15^** |
|  | *C. niger complex* | -0.65 | (-0.89, -0.41) | 0.05 | 8.65 | 169 | **2.01 x 10^-13^** |
|  | *T. graeca* | 1.56 | (1.43, 1.69) | 0.02 | 37.7 | 3961 | **2.3 x 10^-8^** |
|  | *T. hermanni* | 0.11 | (0.03, 0.19) | 0.01 | 4.34 | 8938 | **4.84 x 10^-4^** |
|  | *T. marginata* | 1.59 | (1.36, 1.82) | 0.05 | 21.2 | 428 | **0** |
|  | *A. radiata* | -1.43 | (-2.08, -0.78) | 0.14 | 7.09 | 81.5 | **1.54 x 10^-8^** |
| *C. denticulata* | *I. elongata* | 1.92 | (1.42, 2.43) | 0.11 | 11.9 | 329 | **5.09 x 10^-13^** |
|  | *C. niger complex* | -3.09 | (-3.49, -2.68) | 0.09 | 23.8 | 603 | **4.29 x 10^-10^** |
|  | *T. graeca* | -0.87 | (-1.22, -0.52) | 0.08 | 7.71 | 595 | **4.78 x 10^-10^** |
|  | *T. hermanni* | -2.31 | (-2.65, -1.98) | 0.07 | 21.3 | 505 | **1.61 x 10^-10^** |
|  | *T. marginata* | -0.83 | (-1.24, -0.43) | 0.09 | 6.49 | 802 | **5.39 x 10^-9^** |
|  | *A. radiata* | -3.86 | (-4.58, -3.14) | 0.16 | 17.0 | 130 | **6.33 x 10^-14^** |
| *I. elongata* | *C. niger complex* | -5.01 | (-5.46, -4.56) | 0.10 | 34.9 | 212 | **0** |
|  | *T. graeca* | -2.79 | (-3.20, -2.39) | 0.09 | 21.7 | 152 | **9.64 x 10^-14^** |
|  | *T. hermanni* | -4.24 | (-4.63, -3.84) | 0.08 | 34.0 | 134 | **1.39 x 10^-14^** |
|  | *T. marginata* | -2.76 | (-3.21, -2.31) | 0.10 | 19.3 | 223 | **0** |
|  | *A. radiata* | -5.78 | (-6.53, -5.04) | 0.16 | 24.5 | 139 | **0** |
| *C. niger complex* | *T. graeca* | 2.22 | (1.95, 2.48) | 0.06 | 26.0 | 264 | **6.63 x 10^-14^** |
|  | *T. hermanni* | 0.77 | (0.52, 1.02) | 0.05 | 9.79 | 196 | **1.08 x 10^-13^** |
|  | *T. marginata* | 2.25 | (1.92, 2.58) | 0.07 | 21.3 | 459 | **0** |
|  | *A. radiata* | -0.77 | (-1.46, -0.09) | 0.15 | 3.60 | 104 | **0.01** |
| *T. graeca* | *T. hermanni* | -1.44 | (-1.59, -1.30) | 0.03 | 31.1 | 5845 | **1.86 x 10^-13^** |
|  | *T. marginata* | 0.03 | (-0.23, 0.29) | 0.05 | 0.38 | 671 | 1.0 |
|  | *A. radiata* | -2.99 | (-3.64, -2.34) | 0.14 | 14.6 | 87.4 | **3.29 x 10^-10^** |
| *T. hermanni* | *T. marginata* | 1.48 | (1.23, 1.72) | 0.05 | 18.9 | 497 | **1.21 x 10^-10^** |
|  | *A. radiata* | -1.55 | (-2.19, -0.90) | 0.14 | 7.62 | 83.3 | **1.42 x 10^-9^** |
| *T. marginata* | *A. radiata* | -3.02 | (-3.70, -2.34) | 0.15 | 14.1 | 104 | **3.60 x 10^-14^** |

**Table S9:** Two-sample Wilcoxon rank sum tests comparisons of experienced versus native microclimate temperatures (sun, shade, and sun and shade combined) during all times of day by species. P-values in bold represent statistically significant (< 0.05). (Mean difference = experienced temperature avg – native microclimate avg (sun, shade or both), CI = confidence interval). *C. niger complex* was not included in this analysis as only two individuals could be studied.

| Species | Native Microclimate | Mean difference (^o^C) | 95% CI (Lower, Upper) | W | p-value |
| --- | --- | --- | --- | --- | --- |
| *C. denticulata* | Sun | -1.94 | (-3.83, -1.28) | 998 | **0.002** |
|  | Shade | 0.51 | (-1.23, 1.12) | 2198 | 0.82 |
|  | Both | -0.71 | (-2.52, -0.10) | 1410 | **0.03** |
| *A. radiata* | Sun | 4.84 | (3.69, 5.55) | 820 | **2.79 x 10^-7^** |
|  | Shade | 6.62 | (5.60, 6.80) | 820 | **2.79 x 10^-7^** |
|  | Both | 5.73 | (4.65, 6.17) | 820 | **2.79 x 10^-7^** |
| *C. carbonaria* | Sun | -1.57 | (-2.67, -0.99) | 1187 | **8.19 x 10^-4^** |
|  | Shade | 0.89 | (-0.47, 1.46) | 3568 | 0.40 |
|  | Both | -1.16 | (-2.46, -0.42) | 1718 | **0.01** |
| *I. elongata* | Sun | -2.02 | (-2.94, -1.49) | 106 | **1.96 x 10^-4^** |
|  | Shade | -0.04 | (-1.02, 0.53) | 408 | 0.38 |
|  | Both | -1.03 | (-1.95, -0.45) | 231 | **0.01** |
| *T. graeca* | Sun | -1.83 | (-3.48, -0.17) | 5105 | **0.03** |
|  | Shade | 1.68 | (0.26, 2.93) | 15788 | **0.01** |
|  | Both | -0.07 | (-1.60, 1.40) | 9416 | 0.75 |
| *T. marginata* | Sun | -0.40 | (-1.40, 0.57) | 1755 | 0.43 |
|  | Shade | 3.03 | (2.25, 3.59) | 4072 | **9.81 x 10^-8^** |
|  | Both | 1.31 | (0.46, 2.09) | 3159 | **0.003** |
| *T. hermanni* | Sun | 0.59 | (-0.25, 1.41) | 38787 | 0.12 |
|  | Shade | 3.39 | (2.45, 4.17) | 59266 | **1.48 x 10^-6^** |
|  | Both | 1.99 | (1.33, 2.76) | 50363 | **2.69 x 10^-4^** |
| *T. carolina* | Sun | -2.82 | (-3.78, -1.92) | 7766 | **5.59 x 10^-5^** |
|  | Shade | -0.05 | (-0.96, 0.88) | 61747 | 0.84 |
|  | Both | -1.44 | (-2.35, -0.52) | 24593 | **0.004** |

**Table S10:** Two-sample t-test comparisons of experienced temperatures and enclosure temperatures (sun or shade) during all times of day for each species. P-values in bold represent statistical significance (<0.05). (Mean difference (sun) = sun enclosure temperature avg – experienced temperature avg, Mean difference (shade) = experienced temperature avg – shade enclosure temperature avg, CI = confidence interval, SE = standard error, df = degrees of freedom).

| Species | Enclosure Microclimate | Mean difference (^o^C) | 95% CI (Lower, Upper) | SE (^o^C) | df | t-value | p-value |
| --- | --- | --- | --- | --- | --- | --- | --- |
| *T. carolina* | Sun | 2.13 | (1.53, 2.74) | 0.24 | 6 | 8.66 | **6.53 x 10^-5^** |
|  | Shade | 0.76 | (0.15, 1.36) | 0.24 | 6 | 3.08 | **0.01** |
| *C. denticulata* | Sun | -1.40 | (-3.05, 0.24) | 0.72 | 9 | -1.92 | 0.95 |
|  | Shade | 1.53 | (-0.11, 3.18) | 0.72 | 9 | 2.10 | **0.03** |
| *A. radiata* | Sun | -3.96 | (-4.27, -3.64) | 0.13 | 9 | -28.30 | 1.0 |
| *C. carbonaria* | Sun | -2.95 | (-3.41, -2.49) | 0.20 | 9 | -14.60 | 0.99 |
|  | Shade | 1.70 | (1.25, 2.16) | 0.20 | 9 | 8.45 | **7.08 x 10^-6^** |
| *I. elongata* | Sun | 1.21 | (0.47, 1.95) | 0.31 | 7 | 3.86 | **0.003** |
|  | Shade | 2.95 | (2.20, 3.69) | 0.31 | 7 | 9.39 | **1.61 x 10^-5^** |
| *T. graeca* | Sun | -1.53 | (-2.02, -1.03) | 0.19 | 5 | -7.88 | 0.99 |
|  | Shade | 3.34 | (2.84, 3.84) | 0.19 | 5 | 17.23 | **6.01 x 10^-6^** |
| *T. hermanni* | Sun | -3.36 | (-3.80, -2.93) | 0.19 | 9 | -17.43 | 1.0 |
|  | Shade | 3.52 | (3.08, 3.96) | 0.19 | 9 | 18.24 | **1.01 x 10^-8^** |
| *T. marginata* | Sun | -2.01 | (-2.44, -1.58) | 0.19 | 9 | -10.55 | 0.99 |
|  | Shade | 2.70 | (2.26, 3.13) | 0.19 | 9 | 14.14 | **9.40 x 10^-8^** |
| *C. niger complex* | Shade | 6.61 | (1.18, 12.05) | 0.42 | 1 | 15.48 | **0.02** |

**Table S11:** Influence of species, log body mass, and sex on experienced temperatures across (all 9 species) and within species (only *C. carbonaria*, *C. denticulata*, *T. hermanni*, *T. marginata*, and *A. radiata*). Data for all times during the day across each month for the entire season. P-values in bold represent statistical significance (<0.05) (df = degrees of freedom, Res df = residual degrees of freedom, SS =sum of squares, RSS = residual sum of squares, MS = mean squares). *C. niger* complex was included in these analyses.

| Source | df | Res df | SS | RSS | MS | F-value | p-value |
| --- | --- | --- | --- | --- | --- | --- | --- |
| Avg T_pref_ (9 species)        Species        Log Body Mass  Sex | 8  1  1 | 64  71  71 | 176.08  102.6  5.32 | 69.58  143.0  240.34 | 22.01  102.61  5.317 | 20.25  50.93  2.48 | **7.35 x 10^-15^**  **6.59 x 10^-10^**  0.21 |
| Avg T_pref_ (5 species)        Species        Sex        Species:Sex | 4  1  4 | 40  40  40 | 35.91  8.50  7.43 | 44.05  44.05  44.05 | 8.97  8.50  1.85 | 8.15  7.72  1.68 | **6.62 x 10^-5^**  **0.008**  0.17 |
| Avg T_pref_ (5 species)  Species  Log Body Mass  Species: Log Body Mass | 4  1  4 | 40  40  40 | 36.14  15.43  6.37 | 38.18  38.18  38.18 | 9.03  15.43  1.59 | 9.46  16.16  1.66 | **1.76 x 10^-5^**  **0.0002**  0.17 |

**Table S12:** Tukey post-hoc pairwise species comparisons for “active” experienced temperatures (6 am to 9 pm) across each month for the entire season. P-values in bold represent statistical significance (<0.05).

| Species 1 | Species 2 | Mean difference (^o^C) | 95% CI (Lower, Upper) | p-value |
| --- | --- | --- | --- | --- |
| *C. carbonaria* | *T. carolina* | -4.37 | (-5.93, -2.80) | **0.00** |
|  | *C. denticulata* | -1.58 | (-3.00, -0.16) | **0.01** |
|  | *I. elongata* | -0.04 | (-1.55, 1.45) | 1.00 |
|  | *C. niger complex* | 1.80 | (-0.65, 4.26) | 0.32 |
|  | *T. graeca* | -0.43 | (-2.07, 1.20) | 0.99 |
|  | *T. hermanni* | -0.27 | (-1.69, 1.14) | 0.99 |
|  | *T. marginata* | 0.77 | (-0.64, 2.19) | 0.71 |
|  | *A. radiata* | 1.79 | (0.37, 3.21) | **0.004** |
| *T. carolina* | *C. denticulata* | 2.78 | (1.22, 4.35) | **1.05 x 10^-5^** |
|  | *I. elongata* | 4.32 | (2.67, 5.96) | **0.00** |
|  | *C. niger complex* | 6.17 | (3.62, 8.17) | **0.00** |
|  | *T. graeca* | 3.93 | (2.16, 5.70) | **0.00** |
|  | *T. hermanni* | 4.09 | (2.53, 5.66) | **0.00** |
|  | *T. marginata* | 5.14 | (3.57, 6.70) | **0.00** |
|  | *A. radiata* | 6.16 | (4.60, 7.73) | **0.00** |
| *C. denticulata* | *I. elongata* | 1.53 | (0.02, 3.04) | **0.04** |
|  | *C. niger complex* | 3.38 | (0.92, 5.84) | **0.001** |
|  | *T. graeca* | 1.14 | (-0.49, 2.78) | 0.39 |
|  | *T. hermanni* | 1.30 | (-0.11, 2.72) | 0.09 |
|  | *T. marginata* | 2.35 | (0.93, 3.77) | **4.85 x 10^-5^** |
|  | *A. radiata* | 3.37 | (1.95, 4.79) | **0.00** |
| *I. elongata* | *C. niger complex* | 1.84 | (-0.66, 4.36) | 0.32 |
|  | *T. graeca* | -0.38 | (-2.10, 1.32) | 0.99 |
|  | *T. hermanni* | -0.22 | (-1.73, 1.28) | 0.99 |
|  | *T. marginata* | 0.81 | (-0.68, 2.32) | 0.71 |
|  | *A. radiata* | 1.84 | (0.33, 3.35) | **0.006** |
| *C. niger complex* | *T. graeca* | -2.32 | (-4.83, 0.35) | 0.14 |
|  | *T. hermanni* | -2.07 | (-4.53, 0.38) | 0.16 |
|  | *T. marginata* | -1.03 | (-3.49, 1.43) | 0.91 |
|  | *A. radiata* | -0.005 | (-2.46, 2.45) | 1.00 |
| *T. graeca* | *T. hermanni* | 0.16 | (-1.47, 1.80) | 0.99 |
|  | *T. marginata* | 1.20 | (-0.43, 2.84) | 0.32 |
|  | *A. radiata* | 2.23 | (0.59, 3.87) | **0.001** |
| *T. hermanni* | *T. marginata* | 1.04 | (-0.37, 2.46) | 0.32 |
|  | *A. radiata* | 2.06 | (0.64, 3.48) | **5.04 x 10^-4^** |
| *T. marginata* | *A. radiata* | 1.02 | (-0.39, 2.44) | 0.34 |

**Figure S1:** Average experienced temperatures for each individual of the nine turtle species during the entire day across the season. Letters shared above each species’ boxplot denote non-significant differences in average experienced temperature. Colors represent the gradient from low (blue) to high (orange) temperatures.

**
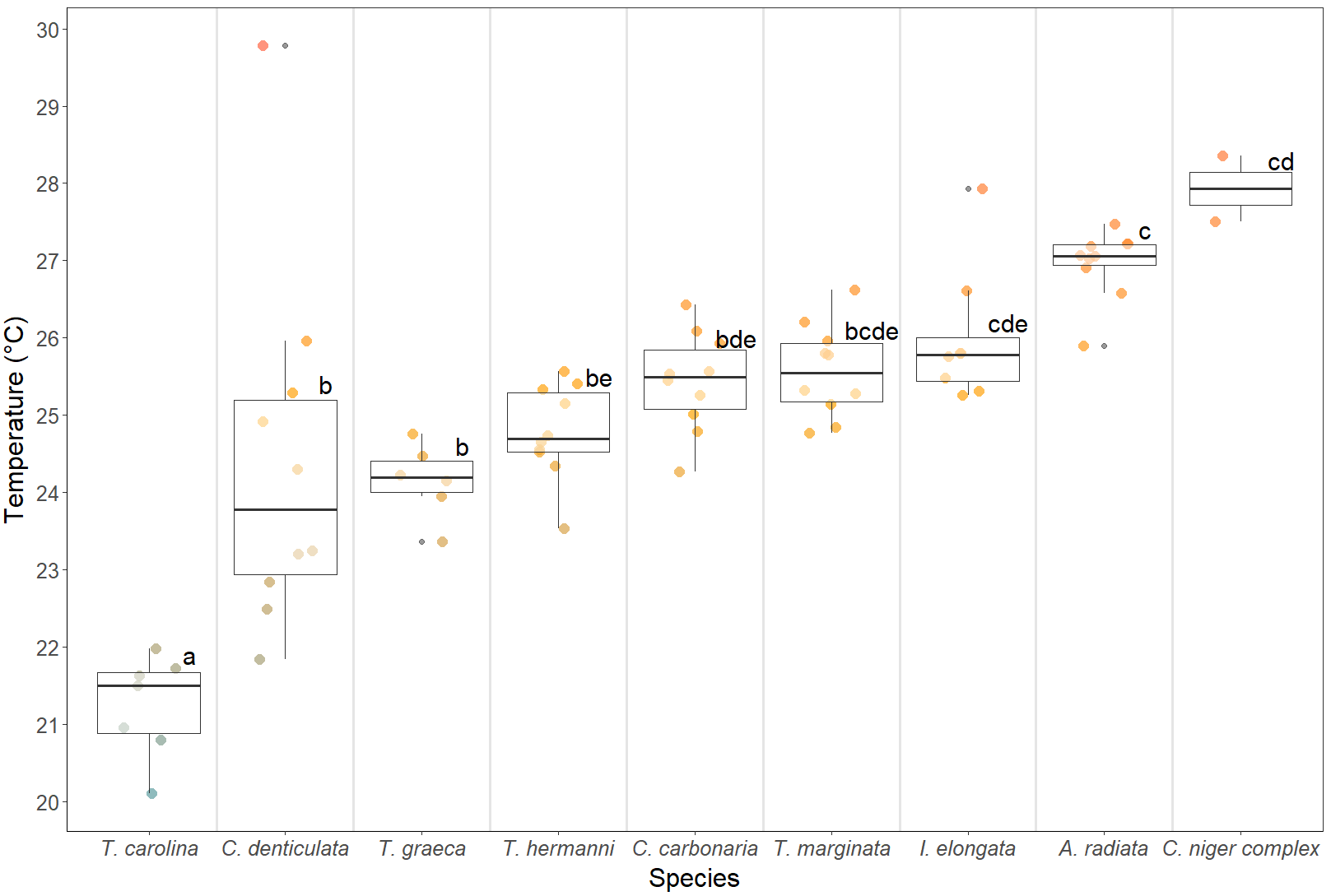
**

**Table S13:** Tukey post-hoc pairwise species comparisons for experienced temperatures for the entire day across all months for the entire season. P-values in bold represent statistical significance (<0.05).

| Species 1 | Species 2 | Mean difference (^o^C) | 95% CI (Lower, Upper) | p-value |
| --- | --- | --- | --- | --- |
| *C. carbonaria* | *T. carolina* | -4.19 | (-5.84, -2.54) | **0.00** |
|  | *C. denticulata* | -1.04 | (-2.54, 0.45) | 0.39 |
|  | *I. elongata* | 0.56 | (-1.02, 2.14) | 0.96 |
|  | *C. niger complex* | 2.49 | (-0.09, 5.09) | 0.06 |
|  | *T. graeca* | -1.27 | (-3.00, 0.45) | 0.31 |
|  | *T. hermanni* | -0.65 | (-2.14, 0.84) | 0.89 |
|  | *T. marginata* | 0.14 | (-1.35, 1.63) | 0.99 |
|  | *A. radiata* | 1.53 | (0.03, 3.02) | **0.04** |
| *T. carolina* | *C. denticulata* | 3.14 | (1.49, 4.79) | **2.1 x 10^-6^** |
|  | *I. elongata* | 4.75 | (3.02, 6.48) | **0.00** |
|  | *C. niger complex* | 6.69 | (4.00, 9.37) | **0.00** |
|  | *T. graeca* | 2.91 | (1.05, 4.77) | **1.43 x 10^-4^** |
|  | *T. hermanni* | 3.54 | (1.89, 5.19) | **1.0 x 10^-7^** |
|  | *T. marginata* | 4.33 | (2.68, 5.98) | **0.00** |
|  | *A. radiata* | 5.72 | (4.07, 7.37) | **0.00** |
| *C. denticulata* | *I. elongata* | 1.60 | (0.01, 3.19) | **0.04** |
|  | *C. niger complex* | 3.54 | (0.94, 6.13) | **0.001** |
|  | *T. graeca* | -0.23 | (-1.96, 1.49) | 0.99 |
|  | *T. hermanni* | 0.39 | (-1.10, 1.88) | 0.99 |
|  | *T. marginata* | 1.18 | (-0.31, 2.68) | 0.23 |
|  | *A. radiata* | 2.57 | (1.07, 4.07) | **2.23 x 10^-5^** |
| *I. elongata* | *C. niger complex* | 1.93 | (-0.70, 4.58) | 0.32 |
|  | *T. graeca* | -1.83 | (-3.64, -0.03) | **0.04** |
|  | *T. hermanni* | -1.21 | (-2.80, 0.37) | 0.27 |
|  | *T. marginata* | -0.41 | (-2.00, 1.16) | 0.99 |
|  | *A. radiata* | 0.96 | (-0.61, 2.55) | 0.57 |
| *C. niger complex* | *T. graeca* | -3.77 | (-6.50, -1.04) | **0.001** |
|  | *T. hermanni* | -3.15 | (-5.74, -0.55) | **0.006** |
|  | *T. marginata* | -2.35 | (-4.94, 0.23) | 0.10 |
|  | *A. radiata* | -0.96 | (-3.55, 1.62) | 0.95 |
| *T. graeca* | *T. hermanni* | 0.62 | (-1.10, 2.35) | 0.96 |
|  | *T. marginata* | 1.41 | (-0.30, 3.14) | 0.19 |
|  | *A. radiata* | 2.80 | (1.08, 4.53) | **7.05 x 10^-5^** |
| *T. hermanni* | *T. marginata* | 0.79 | (-0.70, 2.29) | 0.74 |
|  | *A. radiata* | 2.18 | (0.68, 3.68) | **4.92 x 10^-4^** |
| *T. marginata* | *A. radiata* | 1.38 | (-0.10, 2.88) | 0.08 |

**Table S14:** Influence of species, log body mass, and sex on intraspecific variation (SD) in experienced temperatures across (all 9 species) and within species (only *C. carbonaria*, *C. denticulata*, *T. hermanni*, *T. marginata*, and *A. radiata*). Data for all times during the day across each month for the entire season. P-values in bold represent statistical significance (<0.05) (df = degrees of freedom, Res df = residual degrees of freedom, SS =sum of squares, RSS = residual sum of squares, MS = mean squares). *C. niger* complex was included in these analyses.

| Source | df | Res df | SS | RSS | MS | F-value | p-value |
| --- | --- | --- | --- | --- | --- | --- | --- |
| SD T_pref_ (9 species)        Species        Log Body Mass  Sex | 8  1  1 | 64  71  71 | 32.93  4.03  1.14 | 16.46  45.37  48.25 | 4.11  4.02  1.14 | 16  6.30  1.68 | **1.09 x 10^-12^**  **0.01**  0.21 |
| SD T_pref_ (5 species)        Species        Sex        Species:Sex | 4  1  4 | 40  40  40 | 11.75  0.002  0.82 | 8.41  8.41  8.41 | 2.93  0.001  0.20 | 13.95  0.009  0.97 | **3.26 x 10^-7^**  0.92  0.43 |
| SD T_pref_ (5 species)  Species  Log Body Mass  Species: Log Body Mass | 4  1  4 | 40  40  40 | 6.68  0.003  0.69 | 8.54  8.54  8.54 | 1.67  0.03  0.17 | 7.82  0.01  0.81 | **9.38 x 10^-5^**  0.90  0.52 |

**1. Inter-specific variation in experienced temperatures (across species)**

When analyzing data across the entire day, *T. carolina* had the lowest average experienced temperature (21.23 °C), and the *C. niger* complex had the highest (27.92 °C) (Supp. Fig. S1). Tukey post-hoc tests showed *T. carolina* differed significantly from all other species (p < 0.05), while *C. niger* complex differed from all except *A. radiata* (p = 0.95), *I. elongata* (p = 0.32), *C. carbonaria* (p = 0.06), and *T. marginata* (p = 0.10) (Supp. Fig. S1, Table S13). Significant pairwise differences in the standard deviation of “active” experienced temperatures were found between *C. denticulata*, *T. carolina*, or *I. elongata* and four other species: *T. graeca*, *T. hermanni*, *T. marginata*, and *A. radiata* (p < 0.05) (Supp. Table S15). *C. carbonaria* differed significantly only from *T. graeca* (p < 0.001), *T. hermanni* (p < 0.001), and *T. marginata* (p < 0.001), while *C. niger* complex differed from *T. graeca* (p < 0.001) and *T. hermanni* (p = 0.02) (Table S15). *I. elongata* showed the lowest variation in “active” experienced temperatures (SD = 3.99), and *T. graeca* the highest (SD = 6.16). For the full-day analysis, *T. carolina* and *I. elongata* significantly differed in temperature SD from *T. graeca*, *T. hermanni*, *T. marginata*, and *A. radiata* (p < 0.05), while *C. carbonaria*, *C. denticulata*, and *C. niger* complex showed shared differences with *T. hermanni*, *T. marginata*, and *T. graeca* (Supp. Table S16). *A. radiata* differed significantly from *T. carolina* (p < 0.001), *I. elongata* (p < 0.001), and *T. graeca* (p = 0.01) (Supp. Table S16). Overall, *I. elongata* (mean SD = 3.52) and *T. graeca* (mean SD = 5.59) had the lowest and highest variation in full-day experienced temperatures, respectively (Supp. Fig. S2).

**Table S15:** Tukey post-hoc pairwise species comparisons for variation (SD) in “active” experienced temperatures (6 am to 9 pm) across each month for the entire season. P-values in bold represent statistical significance (<0.05) (CI = confidence interval, Mean difference = Species 2 avg – Species 1 avg).

| Species 1 | Species 2 | Mean difference (^o^C) | 95% CI (Lower, Upper) | p-value |
| --- | --- | --- | --- | --- |
| *C. carbonaria* | *T. carolina* | -0.28 | (-1.01, 0.45) | 0.94 |
|  | *C. denticulata* | -0.02 | (-0.68, 0.64) | 1.00 |
|  | *I. elongata* | -0.61 | (-1.32, 0.09) | 0.14 |
|  | *C. niger complex* | -0.0008 | (-1.157, -1.155) | 1.00 |
|  | *T. graeca* | 1.56 | (0.79, 2.33) | **5.0 x 10^-7^** |
|  | *T. hermanni* | 1.22 | (0.56, 1.89) | **5.1 x 10^-6^** |
|  | *T. marginata* | 0.82 | (0.15, 1.49) | **0.005** |
|  | *A. radiata* | 0.66 | (-0.002, 1.33) | 0.05 |
| *T. carolina* | *C. denticulata* | 0.26 | (-0.47, 0.99) | 0.96 |
|  | *I. elongata* | -0.33 | (-1.10, 0.44) | 0.90 |
|  | *C. niger complex* | 0.28 | (-0.91, 1.47) | 0.99 |
|  | *T. graeca* | 1.84 | (1.01, 2.67) | **0.00** |
|  | *T. hermanni* | 1.51 | (0.77, 2.24) | **3.0 x 10^-7^** |
|  | *T. marginata* | 1.10 | (0.37, 1.84) | **2.94 x 10^-4^** |
|  | *A. radiata* | 0.94 | (0.21, 1.68) | **0.003** |
| *C. denticulata* | *I. elongata* | -0.59 | (-1.30, 0.11) | 0.17 |
|  | *C. niger complex* | 0.02 | (-1.13, 1.17) | 1.00 |
|  | *T. graeca* | 1.58 | (0.81, 2.35) | **3.0 x 10^-7^** |
|  | *T. hermanni* | 1.24 | (0.58, 1.91) | **3.4 x 10^-6^** |
|  | *T. marginata* | 0.84 | (0.17, 1.51) | **0.004** |
|  | *A. radiata* | 0.68 | (0.01, 1.35) | **0.03** |
| *I. elongata* | *C. niger complex* | 0.61 | (-0.56, 1.79) | 0.76 |
|  | *T. graeca* | 2.17 | (1.37, 2.98) | **0.00** |
|  | *T. hermanni* | 1.84 | (1.13, 2.55) | **0.00** |
|  | *T. marginata* | 1.43 | (0.73, 2.14) | **4.0 x 10^-7^** |
|  | *A. radiata* | 1.28 | (0.57, 1.98) | **7.6 x 10^-6^** |
| *C. niger complex* | *T. graeca* | 1.56 | (0.34, 2.78) | **0.003** |
|  | *T. hermanni* | 1.22 | (0.07, 2.38) | **0.02** |
|  | *T. marginata* | 0.82 | (-0.33, 1.98) | 0.36 |
|  | *A. radiata* | 0.66 | (-0.48, 1.82) | 0.64 |
| *T. graeca* | *T. hermanni* | -0.33 | (-1.10, 0.43) | 0.89 |
|  | *T. marginata* | -0.74 | (-1.51, 0.02) | 0.06 |
|  | *A. radiata* | -0.89 | (-1.67, -0.12) | **0.01** |
| *T. hermanni* | *T. marginata* | -0.40 | (-1.07, 0.26) | 0.58 |
|  | *A. radiata* | -0.56 | (-1.23, 0.10) | 0.16 |
| *T. marginata* | *A. radiata* | -0.15 | (-0.82, 0.50) | 0.99 |

**Table S16:** Tukey post-hoc pairwise species comparisons for variation (SD) in experienced temperatures for the entire day across all months for the entire season. P-values in bold represent statistical significance (<0.05) (CI = confidence interval, Mean difference = Species 2 avg – Species 1 avg).

| Species 1 | Species 2 | Mean difference (^o^C) | 95% CI (Lower, Upper) | p-value |
| --- | --- | --- | --- | --- |
| *C. carbonaria* | *T. carolina* | -0.28 | (-1.08, 0.51) | 0.96 |
|  | *C. denticulata* | 0.05 | (-0.67, 0.78) | 0.99 |
|  | *I. elongata* | -0.46 | (-1.23, 0.30) | 0.59 |
|  | *C. niger complex* | -0.45 | (-1.71, 0.81) | 0.96 |
|  | *T. graeca* | 1.61 | (0.76, 2.45) | **2.0 x 10^-6^** |
|  | *T. hermanni* | 1.28 | (0.55, 2.01) | **1.28 x 10^-5^** |
|  | *T. marginata* | 0.87 | (0.14, 1.60) | **8.07 x 10^-3^** |
|  | *A. radiata* | 0.66 | (-0.06, 1.39) | 0.10 |
| *T. carolina* | *C. denticulata* | 0.34 | (-0.46, 1.14) | 0.90 |
|  | *I. elongata* | -0.17 | (-1.02, 0.66) | 0.99 |
|  | *C. niger complex* | -0.16 | (-1.47, 1.13) | 0.99 |
|  | *T. graeca* | 1.89 | (0.98, 2.80) | **2.0 x 10^-7^** |
|  | *T. hermanni* | 1.56 | (0.76, 2.37) | **1.2 x 10^-6^** |
|  | *T. marginata* | 1.15 | (0.35, 1.95) | **6.06 x 10^-4^** |
|  | *A. radiata* | 0.94 | (0.14, 1.75) | **0.009** |
| *C. denticulata* | *I. elongata* | -0.51 | (-1.29, 0.25) | 0.44 |
|  | *C. niger complex* | -0.50 | (-1.76, 0.75) | 0.93 |
|  | *T. graeca* | 1.55 | (0.71, 2.39) | **4.5 x 10^-6^** |
|  | *T. hermanni* | 1.22 | (0.50, 1.95) | **3.30 x 10^-5^** |
|  | *T. marginata* | 0.81 | (0.08, 1.54) | **0.01** |
|  | *A. radiata* | 0.60 | (-0.11, 1.33) | 0.17 |
| *I. elongata* | *C. niger complex* | 0.01 | (-1.27, 1.29) | 1.00 |
|  | *T. graeca* | 2.07 | (1.19, 2.95) | **0.00** |
|  | *T. hermanni* | 1.74 | (0.97, 2.52) | **0.00** |
|  | *T. marginata* | 1.33 | (0.56, 2.10) | **2.00 x 10^-5^** |
|  | *A. radiata* | 1.12 | (0.35, 1.90) | **4.80 x 10^-4^** |
| *C. niger complex* | *T. graeca* | 2.06 | (0.73, 3.39) | **1.70 x 10^-4^** |
|  | *T. hermanni* | 1.73 | (0.47, 2.99) | **0.001** |
|  | *T. marginata* | 1.32 | (0.06, 2.58) | **0.03** |
|  | *A. radiata* | 1.11 | (-0.14, 2.37) | 0.12 |
| *T. graeca* | *T. hermanni* | -0.32 | (-1.16, 0.51) | 0.94 |
|  | *T. marginata* | -0.73 | (-1.57, 0.10) | 0.12 |
|  | *A. radiata* | -0.94 | (-1.78, -0.10) | **0.01** |
| *T. hermanni* | *T. marginata* | -0.41 | (-1.14, 0.31) | 0.66 |
|  | *A. radiata* | -0.62 | (-1.34, 0.10) | 0.15 |
| *T. marginata* | *A. radiata* | -0.20 | (-0.93, 0.52) | 0.99 |

**
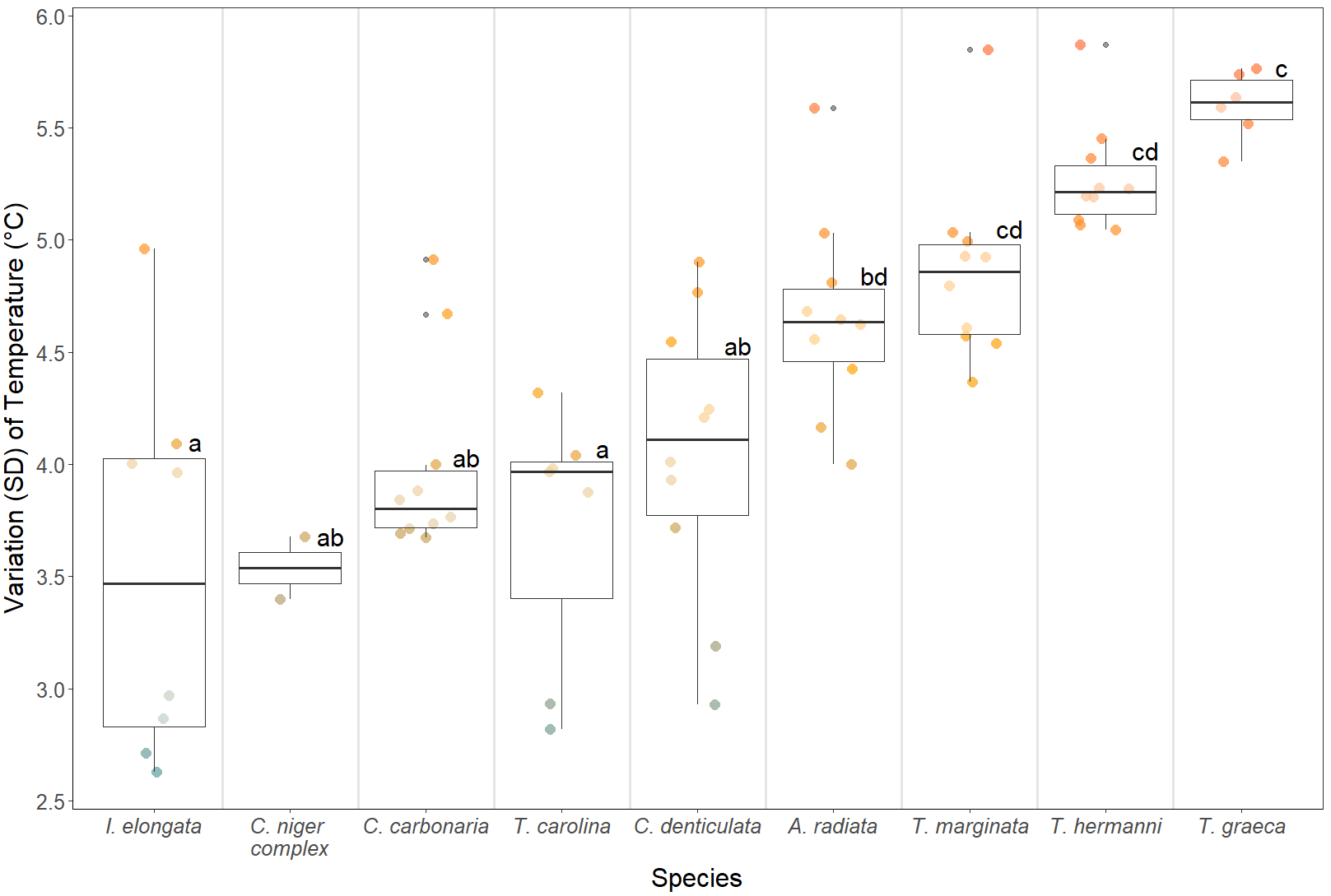
**

**Figure S2:** Variation (SD) of experienced temperatures across nine turtle species for each individual for the entire day across the entire season. Letters shared above each species’ boxplot denote non-significant differences in standard deviations of experienced temperature. Letters not shared between species’ box plots denote significant differences in standard deviations of experienced temperature. Colors represent the gradient from low (yellow) to higher (orange) variation in experienced temperatures.

**2. Intra-specific variation in experienced temperatures (within species)**

Within species, Tukey post-hoc tests showed no significant differences in standard deviation of “active” experienced temperature between male and female individuals for *C. carbonaria*, *C. denticulata*, *T. hermanni*, *T. marginata*, and *A. radiata* (p-values > 0.05, Supp. Materials Table S19). These results were confirmed when using data for the entire day (Supp. Materials Table S20).

**Table S17:** Influence of sex on variation (SD) in experienced temperatures within species. Data only for “active” temperatures (6am to 9pm) across each month for the entire season. Only species for which males and females were sampled and species with 10 individuals were included in the analysis. Average SD of “active” experienced temperature values for each sex calculated from SD values of “active” average experienced values for each individual for each time interval during the day across the season. P-values in bold represent statistical significance (<0.05) (CI = confidence interval, Mean difference = male SD avg – female SD avg).

| Species | Mean SD of temp. for males | Mean SD of temp. for females | Mean difference | 95% CI (Lower, Upper) | p-value |
| --- | --- | --- | --- | --- | --- |
| *C. carbonaria* | 4.34 | 4.71 | -0.37 | (-1.37, 0.61) | 0.95 |
| *C. denticulata* | 4.71 | 4.52 | 0.19 | (-0.80, 1.18) | 0.99 |
| *T. hermanni* | 5.89 | 5.77 | 0.11 | (-0.79, 1.02) | 0.99 |
| *T. marginata* | 5.22 | 5.51 | -0.29 | (-1.28, 0.70) | 0.99 |
| *A. radiata* | 5.21 | 5.32 | -0.11 | (-1.02, 0.79) | 0.99 |

**Table S18:** Influence of sex on variation (SD) in experienced temperatures within species. Data for all times during the day across each month for the entire season. Only species with 10 individuals and for which males and females were sampled were included in the analysis. Average SD of experienced temperature values for each sex calculated from SD of experienced values for each individual for each time interval during the day across the season. P-values in bold represent statistical significance (<0.05) (CI = confidence interval, Mean difference = male SD avg – female SD avg).

| Species | Mean SD of temp. for males | Mean SD of temp. for females | Mean difference | 95% CI (Lower, Upper) | p-value |
| --- | --- | --- | --- | --- | --- |
| *C. carbonaria* | 3.82 | 4.06 | -0.23 | (-1.29, 0.81) | 0.99 |
| *C. denticulata* | 4.37 | 3.90 | 0.47 | (-0.58, 1.53) | 0.88 |
| *T. hermanni* | 5.35 | 5.18 | 0.16 | (-0.80, 1.14) | 0.99 |
| *T. marginata* | 4.81 | 4.88 | -0.06 | (-1.12, 0.99) | 1.00 |
| *A. radiata* | 4.52 | 4.77 | -0.24 | (-1.21, 0.72) | 0.99 |
